# Chemical Compositional Changes in Over-Oxidized Fish Oils

**DOI:** 10.3390/foods9101501

**Published:** 2020-10-20

**Authors:** Austin S. Phung, Gerard Bannenberg, Claire Vigor, Guillaume Reversat, Camille Oger, Martin Roumain, Jean-Marie Galano, Thierry Durand, Giulio G. Muccioli, Adam Ismail, Selina C. Wang

**Affiliations:** 1 Department of Chemistry, University of California, Davis, CA 95616, USA; asphung@ucdavis.edu; 2 Global Organization for EPA and DHA Omega-3s (GOED), Salt Lake City, UT 84105, USA; adam.ismail@kdpharmagroup.com; 3 Institut des Biomolécules Max Mousseron (IBMM), UMR 5247, CNRS, Université de Montpellier, ENSCM, 34093 Montpellier, France; claire.vigor@umontpellier.fr (C.V.); guillaume.reversat@umontpellier.fr (G.R.); camille.oger@umontpellier.fr (C.O.); jean-marie.galano@umontpellier.fr (J.-M.G.); thierry.durand@umontpellier.fr (T.D.); 4 Louvain Drug Research Institute, Université Catholique de Louvain, 1200 Brussels, Belgium; martin.roumain@uclouvain.be (M.R.); giulio.muccioli@uclouvain.be (G.G.M.); 5 Department of Food Science and Technology, University of California, Davis, CA 95616, USA

**Keywords:** dietary supplement, docosahexaenoic acid, eicosapentaenoic acid, fatty acids, food analysis, fish oils, isoprostanoids, omega-3, oxidation condition, oxysterols, volatiles

## Abstract

A recent study has reported that the administration during gestation of a highly rancid hoki liver oil, obtained by oxidation through sustained exposure to oxygen gas and incident light for 30 days, causes newborn mortality in rats. This effect was attributed to lipid hydroperoxides formed in the omega-3 long-chain polyunsaturated fatty acid-rich oil, while other chemical changes in the damaged oil were overlooked. In the present study, the oxidation condition employed to damage the hoki liver oil was replicated, and the extreme rancidity was confirmed. A detailed analysis of temporal chemical changes resulting from the sustained oxidative challenge involved measures of eicosapentaenoic acid/docosahexaenoic acid (EPA/DHA) omega-3 oil oxidative quality (peroxide value, *para*-anisidine value, total oxidation number, acid value, oligomers, antioxidant content, and induction time) as well as changes in fatty acid content, volatiles, isoprostanoids, and oxysterols. The chemical description was extended to refined anchovy oil, which is a more representative ingredient oil used in omega-3 finished products. The present study also analyzed the effects of a different oxidation method involving thermal exposure in the dark in contact with air, which is an oxidation condition that is more relevant to retail products. The two oils had different susceptibility to the oxidation conditions, resulting in distinct chemical oxidation signatures that were determined primarily by antioxidant protection as well as specific methodological aspects of the applied oxidative conditions. Unique isoprostanoids and oxysterols were formed in the over-oxidized fish oils, which are discussed in light of their potential biological activities.

## 1. Introduction

Fish oils are a good source of the omega-3 long chain polyunsaturated fatty acids (PUFAs) (omega-3 LCPUFA), eicosapentaenoic acid (EPA), and docosahexaenoic acid (DHA). The intake of EPA and DHA from the consumption of fish, or fish oils and concentrates (oils containing EPA and DHA in higher concentrations), can confer a range of health benefits, such as lowering blood pressure and reducing the risk for heart disease in adults [1,2]. Specific omega-3 LCPUFAs such as DHA also support the development of the brain and visual system of the fetus during pregnancy [3,4,5,6,7,8]. The intake of fish oil can also impart antioxidant effects though the increased expression of antioxidant enzymes, and components in fish oil are associated with antimicrobial effects [9,10]. Given their relatively high content of polyunsaturated fatty acids, fish oils and other EPA/DHA omega-3-containing oils are susceptible to oxidation [11,12]. Fatty acid composition, antioxidant concentrations, oxidative condition(s) (for example temperature and light), and specific compositional aspects such as trace metal ions and moisture all affect the oxidation rate of edible LCPUFA-rich oils [13,14,15,16]. The handling of fish oils in a correct manner during processing and employing a proper product formulation designed for the applicable storage conditions can effectively prevent oxidation during their production and shelf-life [17]. However, the uncontrolled oxidation of highly unsaturated oils will lead to the formation of an array of oxidation products. Additionally, it is expected that widely dissimilar and unique profiles of oxidation products will be present in distinct fish oils exposed to different oxidizing conditions.

Quality guidelines for edible oils set by regulatory authorities exist to avoid rancid products entering the market. Producers of oils and retailers have also set voluntary quality limits through organizations such as the Global Organization for EPA and DHA Omega-3s (GOED) [18]. Industry guidelines assist in making sure products comply with the applicable quality limits set by regulatory agencies for the markets where products are sold. Before fish oil products are sold to consumers, the oxidative quality of EPA/DHA omega-3 ingredient oils and finished products is typically measured using peroxide value (PV), *para*-anisidine value (p-AV), and total oxidation number (TOTOX). Recent studies show that the large majority of ingredient oils as well as finished products found in retail stores is of acceptable quality, although further improvements in oxidative quality can be made for some retail products [19,20,21,22,23,24,25].

The consumption of fish oils that are mildly oxidized (i.e., close to or just above regulatory limits) has not been found to alter a range of biochemical parameters in the body [26,27]. It is when severely oxidized, rancid oils that surpass quality limits many-fold are administered to experimental animals in supra-normal doses that adverse effects have been observed [28,29]. However, it is important to understand which chemical changes occur in over-oxidized oils in order to evaluate the risk pertaining to the consumption of specific substances that may be formed during oxidation. Risks associated with specific hazardous oxidation products should be studied and discussed in the proper context regarding their occurrence in finished products, product availability to consumers, and the actual exposure, i.e., the volumes (doses) of oil that people ingest.

One specific study published in 2016 that aimed at evaluating the effects of oxidized fish oil on the gestational and perinatal health of rats employed an excessively over-oxidized fish oil [28]. Daily gavage with a high dose of the rancid hoki liver oil throughout gestation led to high newborn mortality, and continued feeding of dams with the damaged oil augmented maternal insulin resistance [28]. The experimental conditions used to create the over-oxidized hoki liver oil involved continuous exposure to oxygen gas over a 30-day period under a fluorescent lamp at room temperature. This approach to create rancid oil does not realistically reflect even the poorest storage conditions of any fish oil retail product. Although the results did not apply to the human consumption of fish oil, statements made in the publication may have created unnecessary bias toward the perceived poor quality of fish oil products on the market, as well as dissuaded women from consuming good quality fish oil supplements during pregnancy. Furthermore, the authors assumed that the developmental disruption and effects on a mother’s metabolism were caused by the oxidation of omega-3 fatty acids in the oil. However, fish oils also contain other fatty acids and lipids that are susceptible to oxidation and that could potentially contribute to the observed outcome. A major newspaper perpetuated the study’s conclusion by stating, “Giving pregnant rats the unoxidized fish oil did not increase mortality rates in their babies, indicating that the lethal effect on newborns came from the chemicals that omega-3 fatty acids break down into during oxidation“ [30]. This conclusion is premature due to the lack of detailed analysis of the composition of the over-oxidized oil.

The objective of the present study was to evaluate the chemical compositional changes occurring during the over-oxidation of hoki liver oil under conditions that very closely replicate those employed in the study by Albert et al. [28]. The results may also provide new insight into the formation of specific oxidation products that could have contributed to the observed developmental and maternal toxicity. In order to determine if the observations could be relevant to other fish oils, refined anchovy oil was also exposed to the same conditions. Anchovy oil is a much more commonly used omega-3 ingredient oil globally for finished products consumed by adults, including during pregnancy [31]. Furthermore, to learn if oil type-specific susceptibility to rancidity development and the associated chemical changes would be distinct under different oxidation conditions, both the hoki liver oil and the anchovy oil were also submitted to thermal oxidation in the absence of light [32]. This condition better reflects the oxidation conditions that may occur in poorly stored finished products, such as encapsulated or bottled fish oils that are further packaged and hence fully protected from light.

The oxidative parameters of edible oils were measured to assess the temporal–chemical changes that occurred over the 30-day period, including PV, p-AV, TOTOX, acid value, oligomers, moisture content, EPA and DHA content, fatty acid composition, tocopherol content, and induction time. This study also focused on understanding changes in the levels of specific classes of secondary oxidation products. The reason for this attention is because in the study by Albert and colleagues, a low level of secondary oxidation, i.e., a p-AV of only 4.5, was reported. This value is well within even the strictest quality guidelines on the oxidative quality of edible oils. The oxidation of esterified EPA and DHA is typically accompanied with a substantial formation of secondary oxidation products. In contrast to the relatively high level of primary oxidation that was achieved after 30 days of accelerated oxidation (PV was 48.8 meq O_2_/kg oil), attaining such a low value of *para*-anisidine-reactive fatty aldehydes suggests that a large portion of volatile secondary oxidation products were lost to the incubation. Secondary oxidation products include fatty aldehydes and dialdehydes with relatively low boiling points, which originate from the chain cleavage of fatty acid peroxides formed in primary oxidation. Some of these volatiles contain conjugated aldehydes that are reactive with cellular proteins and can have cytotoxic effects [33,34,35]. However, having attained the reported toxicity with an over-oxidized hoki liver oil with a low content of volatile reactive aldehydes raises the question of whether any other oxidation products could have been formed that are responsible for the observed perinatal outcomes.

Hence, in addition to volatiles, we evaluated the presence in the over-oxidized hoki liver oil and anchovy oil of two other families of secondary oxidation products that have been uncovered in recent years, namely isoprostanoids and structurally similar isofuranoids, and oxysterols. Some members of these families possess potent biological activity, but their presence in fish oils has hardly received attention. Isoprostanoids contain a cyclopentanic framework resembling prostaglandins and encompass the F_2_-isoprostanes (F_2_-IsoPs) derived from arachidonic acid (AA), F_3_-isoprostanes (F_3_-IsoPs) formed from EPA, F_4_-neuroprostanes (F_4_-NeuroPs) formed from DHA, and F_1_-phytoprostanes (F_1_-PhytoPs) formed from α-linolenic acid (ALA) [36,37,38,39]. Another biosynthetically related reported family of cyclic metabolites includes the isofurans, neurofurans, and phytofurans [40,41,42]. Another group of oxidation products with biological activity are oxysterols. Oxysterols arise from the oxidation of cholesterol as well as other sterols, such as phytosterols [43,44,45,46,47]. The oxidation of sterols can occur either at the ring structures or at the side chains, which gives rise to different oxysterol structures. We reasoned that oxysterols might be more prominently present in an oxidized liver oil compared to a fish body oil such as anchovy oil, since the levels of sterols, such as cholesterol, are relatively high in the liver. This study shows that an understanding of temporal changes in chemical composition in hoki liver and anchovy oil during rancidity development can improve the interpretation of in vivo studies carried out with oxidized oils.

## 2. Materials and Methods

### 2.1. Fish Oils

Minimally refined hoki liver oil was obtained from SeaDragon, Nelson, New Zealand. The oil was shipped in food-grade containers with the headspace under carbon dioxide under cooled conditions to the laboratory at the University of California, Davis. The oil was stored in a −20 °C freezer until the start of the incubations. The hoki liver oil contained 66 mg of EPA and 129 mg of DHA per gram oil. Hoki liver oil had an initial PV of 2.0 meq O_2_/kg, p-AV of 12.2, a TOTOX number of 16.2, and an acid value of 1.6 mg KOH/g, according to the manufacturer’s certificate of analysis. The oil did not contain added antioxidants, as in the study by Albert et al. [28], but it still contained some α-tocopherol naturally present in the oil (see Results). Hoki liver oil is an oil extracted from the livers of New Zealand hoki (*Macruronus novaezelandiae*) and minimally refined. New Zealand hoki is also known with the following market names: blue grenadier, whiting, New Zealand hake, and blue hake.

Refined anchovy oil (MEG-3; triglyceride form) was obtained from DSM Nutritional Products Canada Inc., Mulgrave, Nova Scotia, Canada. Per gram of oil, the formulation contained 1.1 mg of tocopherols in 0.5 mg sunflower oil. The anchovy oil was shipped in aluminum bottles under cooled conditions to the laboratory and stored under the same conditions as the hoki liver oil. The oil had an initial PV of 0.0 meq O_2_/kg, p-AV of 5, and TOTOX of 5, according to the manufacturer’s certificate of analysis. The anchovy oil contained 136 mg of EPA and 122 mg of DHA per gram of oil. The natural cholesterol content was 5.29 mg/g. The added tocopherols used for the stabilization of the anchovy oil consisted of “mixed” tocopherols (a mixture of α, β, γ, and δ-tocopherols. This refined and mixed tocopherol-stabilized anchovy oil is reflective of a standard refined fish oil ingredient that is commonly used for consumer products (in encapsulated form or as liquid bottled form). Both the hoki liver oil and anchovy oil were compliant with the quality requirements on environmental contaminants for refined EPA/DHA omega-3 oil ingredients [18].

### 2.2. Oxidation Conditions and Incubations

All incubations involved placing 150 g of oil into a 250 mL pyrex griffin beaker and were performed in triplicate. The main oxidation condition (Condition A) aimed to reproduce as closely as possible the oxidation conditions employed and described by Albert et al. [28]. Oil samples were bubbled continuously with oxygen gas delivered from a gas tank (99.5% oxygen, Praxair, Danbury, CT, USA) for 30 days under standard fluorescent lighting and at room temperature. The beakers were placed in a fumehood with fluorescent lighting (Philips, Cambridge, MA, USA) with a warm white color (wavelength range 400–700 nm). The tops of the beakers were covered with aluminum foil and allowed space for C-flex tubes (Cole-Palmer, Vernon Hills, IL, USA) to deliver oxygen gas to the oils. An acrylic flowmeter (CNBTR LZQ-3, 0-5 LPM, Amazon, Seattle, WN, USA) was set at <0.1 L/min to achieve a flowrate of 1 bubble every 1–2 s.

In order to assess the relative susceptibilities to oxidation of both oils to a different form of oxidation, a second incubation protocol termed Condition B was applied. This condition involved incubating the oils (same quantity and beakers as in condition A) inside an oven at 50 °C with continuous exposure to ambient air (i.e., no bubbling) without incident light [14]. Beakers were wrapped and loosely covered with aluminum foil before being placed in the oven in order to omit any light.

Samples were taken at various time points throughout the 30-day oxidation period: 0, 1, 6, and 12 h, 1, 2, 5, 15, and 30 days. Samples were immediately placed into labeled vials and stored in a −20 °C freezer until analysis. PV, p-AV, acid value, and induction time were measured at all time points. EPA and DHA levels, fatty acid composition, oligomers, tocopherols, volatiles, isoprostanoids, and oxysterols were measured at baseline, 5, 15, and 30 days (using oxidation Condition A).

### 2.3. Primary Indicators of Oxidative Quality

Analysis of PV, p-AV, and acid value was conducted according to the American Oil Chemists’ Society Official Method Cd 8b-90, Cd 18–90 and Ca 5a-40, respectively [48]. The TOTOX number was calculated using the formula 2PV + p-AV [18]. All measurements were carried out in triplicate. Oligomers are oxidatively cross-linked triacylglycerols formed during lipid peroxidation [49]. The extent of oligomerization (expressed as % oligomers) was analyzed by EPAX/Pelagia (Ålesund, Norway) using gel permeation chromatography according to the U.S. and European Pharmacopeia [50]. One or two drops of oil were placed in a 10 mL volumetric flask and dissolved in tetrahydrofuran. Forty μL of the solution was injected, separated on Styragel columns (Waters, Milford, MA, USA), and detected by differential refractometry. The flow rate of the tetrahydrofuran mobile phase was 0.8 mL/min and the run time was 45 min.

### 2.4. Moisture

Moisture content was conducted according to the AOCS Official Method Ca 2c-25 [48]. The limit of detection was 0.001 g of water per five grams of weighed oil.

### 2.5. Induction Time

Induction time was determined using an 892 Professional Rancimat instrument (Metrohm AG, CH-9100 Herisau, Switzerland) according to the Metrohm Rancimat protocol [51]. Induction time measures the length of time it takes until secondary oxidation products begin forming, which is reflected by the point where the oil becomes rancid and displays an increased change in conductivity under experimental conditions that promote the accelerated oxidation of the oil sample.

### 2.6. EPA and DHA

The content of EPA and DHA in the oils was quantified by Alkemist Labs using the USP 40 method “Fats and Fixed Oils, Omega-3 Fatty Acids Determination and Profile <401>” [52]. First, 250 mg of oil was placed into a 5 mL volumetric flask with 4 mL of an antioxidant solution (0.05 mg/mL butylated hydroxytoluene in 2,2,4-trimethylpentane) before being vortexed for 30 s. The flask was filled to volume with the antioxidant solution. Then, 250 mg of oil was placed into another 5 mL volumetric flask, and 4 mL of internal standard (7.0 mg/mL methyl tricosanoate in antioxidant solution) was added. It was vortexed for 30 s and filled to volume. For both solutions, 2 mL was transferred into a 15 mL screw cap vial, and the solvent was evaporated with nitrogen gas. Then, 1.5 mL of a 2% methanolic sodium hydroxide solution was added before being covered with nitrogen, capped, vortexed, and placed in a boiling water bath for 7 min. After cooling, 2 mL of a 12% boron trichoride solution was added and boiled again for 30 min. After cooling to 45 °C, 1 mL of 2,2,4-trimethylpentane and 5 mL of a saturated aqueous sodium chloride were added. It was settled until the organic layer became clear. The organic layer was transferred into a separate tube, and the aqueous layer was mixed with 1 mL of 2,2,4-trimethylpentane. The 2,2,4-trimethylpentane layers were combined and washed twice with 1 mL portions of water. The contents were dried with anhydrous sodium sulfate and transferred to a vial for analysis by gas chromatography using an AP179 CP-Wax 52 CB column (25 m × 0.25 mm × 0.20 μm). The carrier gas, split ratio, and injection volume were hydrogen, 200:1, and 1 μL, respectively. The injector and flame ionization detector temperatures were 250 and 270 °C, respectively. EPA and DHA content were measured in duplicate.

### 2.7. Fatty Acid Classes

The major classes of esterified fatty acids were analyzed in duplicate according to the U.S. Pharmacopeia (USP) 40 method “Fatty Acid Composition <401>” [52]. This official method allows the measurement of the following fatty acids: saturated: C12:0, C14:0, C15:0, C16:0, C17:0, C18:0, C20:0, and C22:0; monounsaturated: C15:1 n-5, C16:1 n-7, C17:1 n-7, C18:1 n-9, C20:1 n-9, and C22:1 n-9; di-unsaturated: C18:2 n-6 and C20:6 n-6; and polyunsaturated: C18:3 n-3, C20:3 n-3, and C22:6 n-3. The analysis was performed by Alkemist Labs (Garden Grove, CA, USA). For each sample, 100 mg of oil was weighed into a 50 mL round-bottom flask. Then, 4 mL of a 0.5 N methanolic sodium hydroxide solution was added before being refluxed for 5 min. Then, 5 mL of a 14% boron trifluoride in methanol solution was added and refluxed for 2 min. Then, 4 mL of heptane was added through a condenser, swirled, and refluxed for 1 min. The mixture was left to cool and transferred into a separatory funnel. Then, 15 mL of a saturated sodium chloride was added. The mixture was shaken, and the layers were allowed to separate. The top heptane layer was collected and dried with 100 mg of sodium sulfate and diluted 10-fold with heptane. The final product was transferred into an HPLC vial for analysis by gas chromatography. Fatty acid methyl esters were separated using an AP195 DB-Wax (30 m × 0.53 mm × 1 μm) column. The carrier gas was hydrogen with a split ratio of 1:1. The injector temperature and injection volume were 220 °C and 1 μL, respectively. The flame-ionization detector temperature was set to 260 °C. Results were expressed in area percentage (area %) as stipulated by the method. Fatty acid composition was measured in duplicate for each sample.

### 2.8. Tocopherols

Alpha-, β- plus γ-tocopherol (β + γ), and δ-tocopherols were measured according to the method of Gimeno et al. [53]. First, 40 μL of oil was placed into a 2 mL Eppendorf tube. Then, 160 μL of hexane was added, after which it was vortexed briefly. Then, 600 μL of methanol and 200 μL of an internal standard solution (tocopherol acetate in ethanol) were added to the tube. The tube was vortexed for 1 min. After centrifugation (5000 rpm, 5 min), the supernatant was filtered through a 0.45 μm Nylon filter into a 2 mL amber vial. Alpha, β + γ, and δ-tocopherols were measured by reversed-phase high performance liquid chromatography (HPLC) and UV detection using an Agilent 1290 Infinity HPLC with autosampler and photodiode array detection. The separation of analytes was achieved on an Agilent ZORBAX Eclipse Plus C_18_ column (3.5 μm pore size, 3 mm inner diameter, 100 mm column length; Agilent Technologies, Santa Clara, CA, USA). The mobile phase was nanopure water and methanol (4:96), and the wavelength for detection was 292 nm. The injection volume was 20 μL and the run time was 10 min.

### 2.9. Volatiles

Five volatiles were chosen for quantification: 1-penten-3-one, (*E*)-2-pentenal, 1-penten-3-ol, (*E*,*E*)-2,4-heptadienal, and (*E*,*Z*)-2,6-nonadienal. These volatile compounds are formed in the oxidation of fish oils, and they have been associated with fishy and rancid off flavor [14,54]. Solid-phase microextraction was used to extract volatile compounds from the oil samples [55]. One gram of oil was weighed into a 20 mL clear glass vial (Agilent Technologies, Santa Clara, CA, USA). Then, 50 μL of a 1/10 dilution of internal standard solution (20 uL of 4-methyl-2-pentanol in 50 mL olive oil) was added to the vial. A small stir bar was added before sealing the vial with a PTFE/silicon septum (Supelco, Bellefonte, PA) and with parafilm. The vial was placed on a heating plate set to 40 °C. After 10 min, a SPME fiber (DVB/CAR/PDMS, Sigma-Aldrich, St. Louis, MO, USA) was inserted into the vial and exposed to the headspace for 45 min. Volatile analysis took place on a Varian 450-GC equipped with a 220-MS ion trap mass spectrometer (Agilent Technologies, Santa Clara, CA, USA). The fiber was injected into the injector port where the volatiles were desorbed. After 5 min, the fiber was removed. The compounds were separated on a Supelcowax 10, fused-silica capillary column (60 m × 0.25 mm × 0.25 μm, Sigma-Aldrich, St. Louis). Helium was used as the carrier gas at a flow rate of 1.5 mL/min. The oven temperature was initially set at 40 °C for 5 min, increased to 150 °C at a rate of 3 °C/min, and then increased to 200 °C at 10 °C/min. Lastly, it was held at 200 °C for 10 min, with a total run time of 56.67 min. The injector temperature was set at 260 °C.

### 2.10. Isoprostanoids

Isoprostanoids were extracted and analyzed at the Institut des Biomolécules Max Mousseron laboratory in Montpellier, France. The compounds were extracted from the oils based on the protocol used in a previous study and corresponded to the non-esterified fraction [56]. One gram of oil was placed into a 50 mL Falcon conical centrifuge tube. Then, 5 mL of hexane, 2 mL of methanol, and 4 μL of a mix of three internal standards (IS) were added. The mixture was incubated for 30 min under agitation at 100 rpm. After 30 min, 2 mL of 20 mM formic acid (pH 4.5) was added. The mixture was vortexed and then centrifuged at 2000 rpm for 5 min at room temperature. The upper phase was discarded before loading the samples into a Solid Phase Extraction 96-well plate (OasisMax 3 cc 60 mg, Waters, Milford, MA, USA). The well plate was conditioned and balanced with 2 mL of methanol and 2 mL of 20 mM formic acid (pH 4.5), respectively. Once samples were loaded, the columns were washed with four successive solutions: 2 mL of a 2% NH_3_ solution, 2 mL of methanol: 20 mM formic acid (30:70 v:v), 2 mL of hexane and then 2 mL of a hexane:ethyl acetate (70:30 v:v). Afterwards, the column was dried under nitrogen for 2–3 min. Then, the samples were eluted with 2 portions of 1 mL of hexane:ethanol:acetic acid (70:29.4:0.6 v:v:v). The solutions were transferred into thermovials and evaporated under nitrogen flux (0.7 bar) at 40 °C for ≈30 min. The sample was solubilized with 100 μL of water:acetonitrile (83:17) and then vortexed. After 15 min, the sample was vortexed again and stored until analysis.

Isoprostanoid analysis was carried out using an Eksigent MicroLC 200 plus (Eksigent Technologies, Dublin, CA, USA) on a HALO C_18_ column (100 × 0.5 mm, 2.7 μm; Eksigent Technologies, Dublin, CA, USA) [57]. The column was kept at 40 °C. The mobile phase used two gradients. Solvent A consisted of 0.1% (*v/v*) formic acid in water, while solvent B consisted of an 8:2 ratio of acetonitrile/methanol with 0.1% (*v/v*) of formic acid. The flowrate was set to 0.03 mL/min. The gradient changes are shown accordingly: 17% solvent B at 0 min until 1.6 min, 21% B at 2.8 min, 25% B at 7.3 min, 28.5% B at 8.8 min, 33.1% B at 9.6 min, 33.3% B at 10.9 min, 40% B at 15 min, and 95% B at 16.5 min for 2.5 min. Mass spectrometric analysis was carried out on an AB Sciex QTrap 5500 (Sciex Applied Biosystems, Concord, ON, Canada) using Electrospray ionization electrospray and operated in negative mode. Source voltage was kept at −4.5 kV and N_2_ was used as the curtain gas. The detection of the ion products from the isoprostanoid deprotonated molecule was performed in the multiple reaction monitoring mode (MRM). The parameters were optimized for each compound. The measured isoprostanoids are shown in Supplementary Figure S2B and S2C. In this method, only non-esterified isoprostanoids present in the oils were measured. Concentration of the analytes was obtained by calibration curves calculated by the area ratio of analytes and IS. Data processing was achieved using the MultiQuant 3.0 software (Sciex Applied Biosystems).

### 2.11. Oxysterols

The extraction and analysis of the free unesterified oxysterols was carried out at the Louvain Drug Research Institute, Université catholique de Louvain in Brussels, Belgium, as described in a previous study [58]. First, 10 or 50 mg of oil was placed into a glass vial that contained dichloromethane:methanol:water (8:4:2 v:v:v). Then, two internal standards, d_7_-4β-hydroxycholesterol (d_7_-4β-OHC) and d_7_-24-hydroxycholesterol (d_7_-24-OHC), were added. To prevent the unwanted formation of oxysterols, 10 μg of butylated hydroxytoluene (BHT) and 20 ng of ethylenediaminetetraacetic acid (EDTA) were added to the mixture. Afterwards, the vials were shaken thoroughly and sonicated in ice-cold water. Then, the samples were centrifuged and the organic layer was recovered and evaporated under nitrogen. Oxysterols were pre-purified by solid-phase extraction using silica as the stationary phase and hexane/isopropanol mixtures as eluents. The oxysterol fraction was eluted and analyzed by HPLC-MS using a LTQ-Orbitrap mass spectrometer coupled to an Accela HPLC system (ThermoFisher Scientific, Waltham, MA, USA) [58]. The following oxysterols were quantified using oxysterol internal standards and calibration curves prepared using the same above procedure: 5α,6α-epoxycholesterol, 5β,6β-epoxycholesterol, 7-hydroxycholesterol, 4β-hydroxycholesterol, 27-hydroxycholesterol, 7α-hydroxycholestenone, 7-ketocholesterol, 25-hydroxycholesterol, and 5α,6β-dihydroxycholesterol.

### 2.12. Data Analysis

The majority of the results are presented as mean ± standard deviation from triplicate measurements. Results from the primary oxidative quality parameters (PV, p-AV, TOTOX number, acid value), as well as moisture, induction time, tocopherols, volatiles, and fatty acid profile, are given as means of three technical replicates of the three parallel incubations of each oil under each oxidation condition. The content of oxysterols and isoprostanes is given as the mean of three technical replicates, and EPA and DHA contents are given as the mean of two technical replicates of one incubation under oxidation Condition A. Oligomer quantification was done with no replications of one incubation under oxidation Condition A. Graphs were made using Windows Excel and SigmaPlot v.13 (Systat Software).

## 3. Results

### 3.1. Peroxide Value

**Figure 1:**
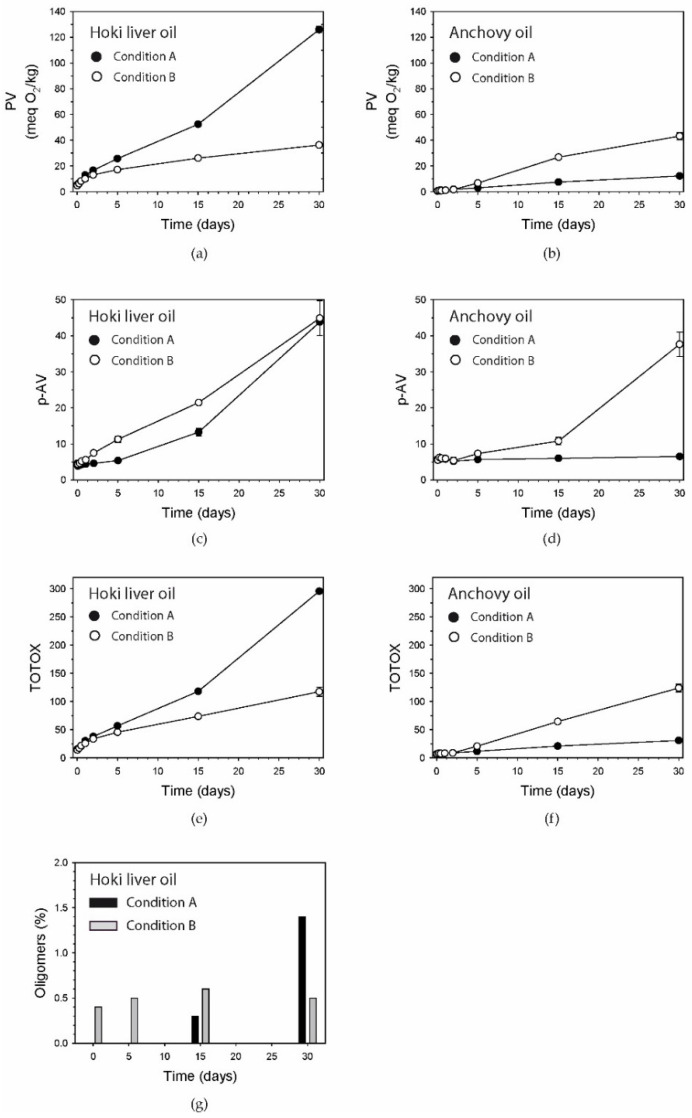
Oxidative changes over a 30-day period of exposure under Conditions A (black circles) and B (open circles): (**a**) peroxide value (PV) of hoki liver oil and (**b**) anchovy oil, (**c**) *para*-anisidine value (p-AV) of hoki liver oil and (**d**) anchovy oil, and (**e**) total oxidation number (TOTOX) number of hoki liver oil and (**f**) anchovy oil. Results are means ± S.D. of three technical replicates for three parallel incubations. (**g**) Oligomer content in hoki liver oil (black bars) and anchovy oil (gray bars) under oxidation Condition A (one single measurement of one incubation shown).

The PV of hoki liver oil gradually increased as a result of continuous exposure to oxygen and light at room temperature (Condition A) (Figure 1a).

After one day, the PV had increased about 7 meq O_2_/kg, and after 30 days, an extremely high level of 126 meq O_2_/kg was reached. In the anchovy oil exposed to the same conditions, notable increases were seen from about five days onwards, with the final level reaching 12.2 meq O_2_/kg (Figure 1b). This photooxidation method had a much larger impact on PV in the hoki liver oil than in the anchovy oil, with a PV at 30 days that was ≈10 times higher. In contrast, the thermal oxidation condition (Condition B) had a similar impact on the PV in both oils (36.3 ± 1.6 meq O_2_/kg in the hoki liver oil and 43.2 ± 2.7 meq O_2_/kg in the anchovy oil; Figure 1a,b). 

### 3.2. p-Anisidine Value

The oxidation of hoki liver oil using both oxidation conditions resulted in similar high p-AVs (43.8 ± 0.3 and 44.9 ± 4.8, respectively) after 30 days of exposure (Figure 1c). Elevations in p-AV started to occur a few days earlier under Condition B than with Condition A. Anchovy oil was sensitive to thermal oxidation, with a marked increase in p-AV occurring in the second half of the incubation period but unaffected under Condition A (Figure 1d). p-AVs in the hoki liver oil and anchovy oil after 30 days of incubation were similar under Condition B.

### 3.3. TOTOX Number

Distinctly different TOTOX values were attained in hoki liver oil and anchovy oil after 30 days of oxidation using Condition A (Figure 1e,f). Hoki liver oil oxidized for 30 days using Condition A had a TOTOX value of 295.7, which was over two times greater than that determined using oxidation Condition B (117.4). The very high TOTOX number for hoki liver oil under Condition A was primarily due to the very high PV. The TOTOX number of anchovy oil after 30 days under Condition B was approximately four times greater than that under Condition A.

### 3.4. Oligomers

Oligomers were not detected in hoki liver oil within the first 5 days of oxidation under Condition A (Figure 1g) but appeared after 15 days. After 30 days, 1.4% of the oil was found as oligomers. The anchovy oil contained 0.4% oligomers, and this level remained relatively constant throughout the incubation.

### 3.5. Acid Value

No changes in acid value (AV) occurred in both oils throughout the 30-day incubation periods under both oxidation conditions. AV consistently remained around 1.5 and 0.5 mg KOH/g in hoki liver oil and in anchovy oil, respectively (Figure S1).

### 3.6. Moisture Content

The moisture content of both hoki liver oil and anchovy oil was below the minimum detectable level (0.001 g water per 5 g of oil) at baseline and throughout the 30-day incubations (data not shown).

### 3.7. Induction Time

**Figure 2:**
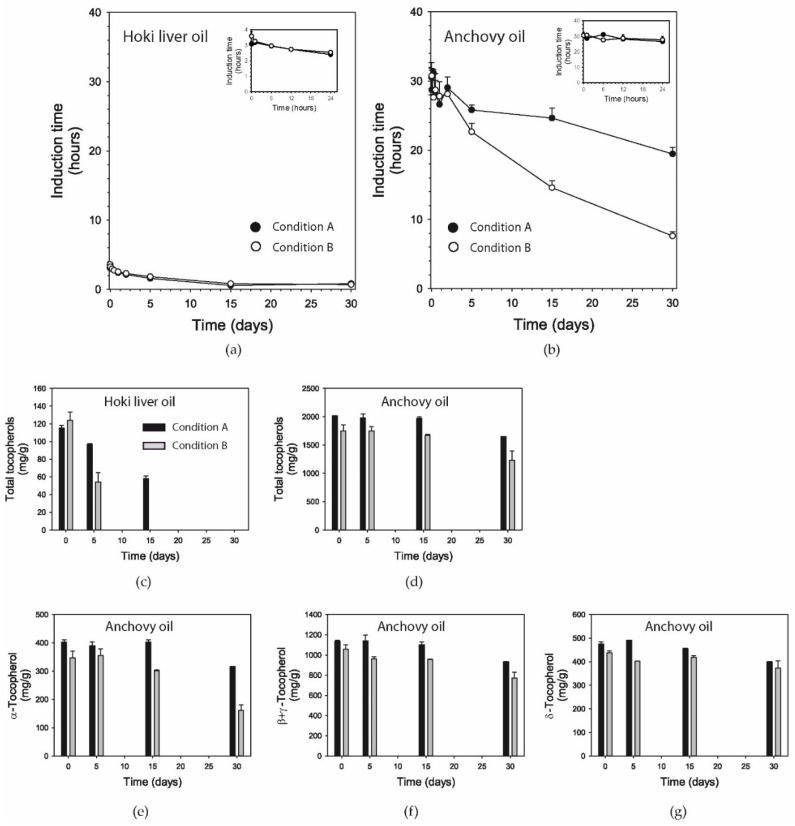
Induction time of (**a**) hoki liver oil and (**b**) anchovy oil over a 30-day period under Conditions A (black symbols) and B (open symbols). Total tocopherol concentrations in (**c**) hoki liver oil and (**d**) anchovy oil over a 30-period under Condition A (black bars) or B (gray bars). The specific tocopherols present in anchovy oil: (**e**) α-tocopherol, (**f**) β- plus γ-tocopherol), and (**g**) δ-tocopherol. In hoki liver oil, only α-tocopherol was present (i.e., the same as total tocopherol shown in panel **c**). Results are means ± S.D. of three technical replicates for three parallel incubations.

Induction time reflects the oxidative stability of dietary oils; i.e., a longer induction time corresponds to higher resistance to oxidation. The hoki liver oil had an induction time of ≈3 h, which started decreasing after several hours of oxidative challenge, with both conditions having approximately the same effect (Figure 2a).

After 30 days, the induction time had decreased to less than one hour. The anchovy oil had an induction time of ≈30 h, i.e., which is ≈10x higher than the hoki liver oil. There were hardly any changes occurring during the first two days of oxidation with both conditions (Figure 2b). The loss of induction time from thereon was faster with Condition B than with Condition A., achieving a reduction by ≈35% (≈20 h) and ≈75% (≈8 h) at 30 days, respectively. At the end of the 30-day oxidation period, the induction time of anchovy oil remained markedly higher than that of hoki liver oil under both conditions.

### 3.8. Tocopherols

Hoki liver oil was measured to contain 115 mg/kg α-tocopherol, and no other tocopherol species were naturally present (Figure 2c). When hoki liver oil was oxidized using Conditions A and B, tocopherol levels had decreased markedly after 5 days. Under Condition A, α-tocopherol was completely oxidized after 30 days, whereas with Condition B, α-tocopherol had become depleted already after 15 days.

In anchovy oil, α, β plus γ, and δ tocopherols were measured to be present at ≈400, ≈1000, and ≈350 mg/kg (Figure 2e–g), respectively. In this oil, which contained approximately a 20-fold higher concentration of total tocopherols than hoki liver oil, the levels of tocopherols remained stable for the first 15 days, followed by a decrease in the second half of the incubation period under both conditions (Figure 2d). The oxidation of α-tocopherol was more marked with the exposure at elevated temperature, but substantial levels of tocopherol still remained after 30 days.

### 3.9. EPA and DHA

**Figure 3:**
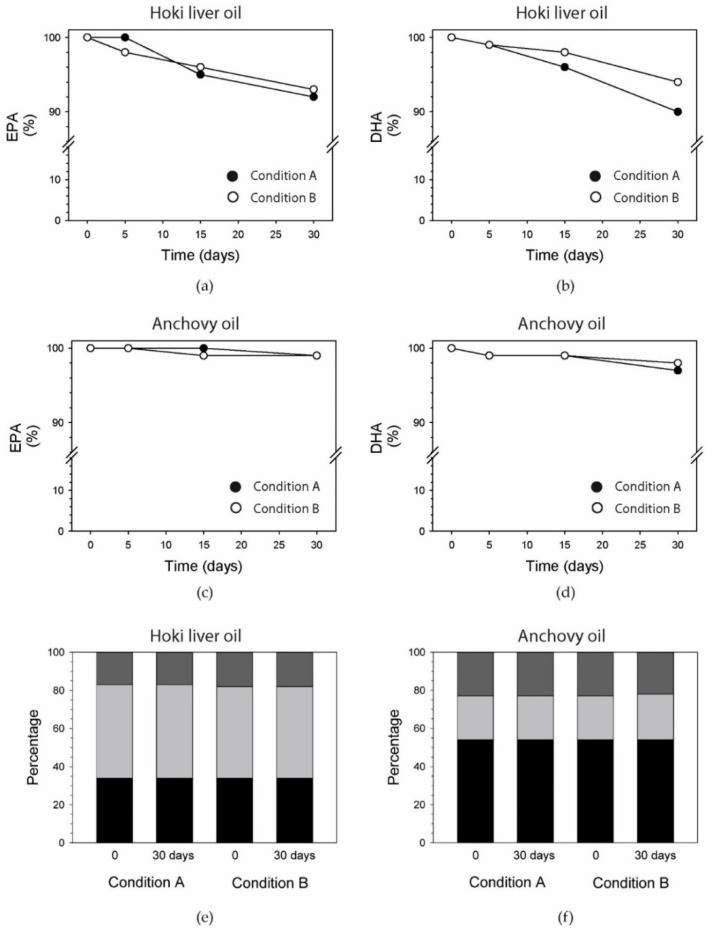
Changes in eicosapentaenoic acid (EPA) and docosahexaenoic acid (DHA) content during the over-oxidation of (**a**,**b**) hoki liver oil and (**c**,**d**) anchovy oil. Hoki liver oil started with 66 mg/g EPA and 129 mg/g DHA. Anchovy oil started with 136 mg/g EPA and 122 mg/g DHA. Results are means ± S.D. of two technical replicates for three parallel incubations. The relative contents of saturated (black bars), monounsaturated (light gray bars), and polyunsaturated fatty acids (dark gray bars) of (**e**) hoki liver oil and (**f**) anchovy oil before and after the 30 days of oxidation under Conditions A and B. Results are means ± S.D. of three technical replicates for three parallel incubations.

The content of the highly unsaturated fatty acids EPA and DHA was determined specifically, using a quantitative method and reported as a function of time. In hoki liver oil there was an 8% and 7% decrease in EPA content after 30 days of oxidation under Conditions A and B, respectively (Figure 3a).

DHA decreased by approximately the same extent (Figure 3b). The decrease in EPA and DHA was lower in anchovy oil; after 30 days, 1% of EPA content and 1% of DHA content had been oxidized under Condition A (Figure 3c), and 3% of EPA and 2% of DHA content had been oxidized under Condition B (Figure 3d).

### 3.10. Fatty Acids

The relative abundances of a number of saturated fatty acids (SFA), monounsaturated fatty acids (MUFA), and polyunsaturated fatty acids (PUFA) were determined to gain a better understanding of the relative susceptibility of both oils to oxidation. To that purpose, several fatty acids were measured using an official US pharmacopeia method (expressed in area %) in both hoki liver oil and anchovy oil over the 30-day period (Table S1). This method did not include several LCPUFA, including arachidonic acid (AA), EPA, and docosapentaenoic acid (DPA) n-3, and hence, it under-reports total PUFA content. Based on the fatty acids that were measured, hoki liver oil had relative proportions of 31.4 ± 0.5% SFA, 48.4 ± 0.7% MUFA, and 17.5 ± 0.2% PUFA, whereas anchovy oil contained 53.9 ± 0.2% SFA, 23.2 ± 0.3% MUFA, and 22.9 ± 0.1% PUFA (mean ± S.D.; *n* = 3) (Figure 3e). Hoki liver oil had a higher content of monounsaturated fat compared to anchovy oil, whereas the opposite was seen for saturated fat. The relative levels of the major fatty acid classes did not change over the course of the incubations (Figure 3f).

The fatty acid with the highest abundance in hoki liver oil was oleic acid (C18:1n9) (≈23%) (Table S1), which matches a previous published study [59]. C16:0 had the second highest abundance (≈16%), which was followed by C22:6n3 (≈12%). The fatty acid with the highest abundance in anchovy oil was palmitic acid (C16:0) (≈18%), which matches the level reported [60]. C22:6n3 had the second highest abundance (≈15%) followed by C22:0 (≈12%) and oleic acid (11%). The levels of the PUFA linoleic acid (≈1% in hoki liver oil and 1.7% in anchovy oil), C18:3 n-6 (≈0.4% in hoki liver oil and 1.0% in anchovy oil), and α-linolenic acid (ALA) (≈0.1% in hoki liver oil and 0.4% in anchovy oil) remained constant throughout the 30 days under both oxidation conditions (Table S1).

### 3.11. Volatiles

**Figure 4:**
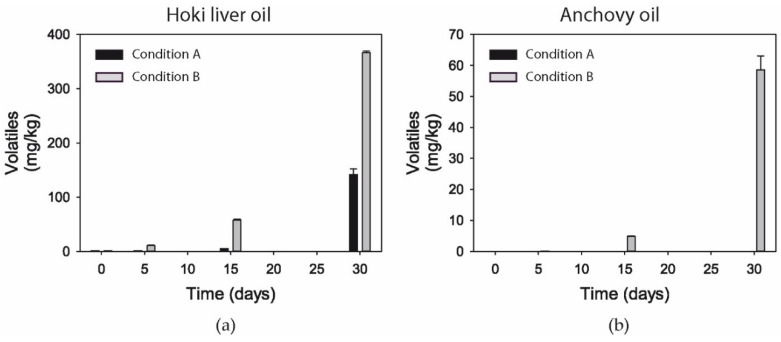
Total concentration of the five volatiles combined in (**a**) hoki liver oil and in (**b**) anchovy oil over a 30-day period under oxidation condition A (black bars) and condition B (gray bars). Results are means ± S.D. of three technical replicates for three parallel incubations.

Volatiles represent a portion of the secondary oxidation products that can be formed from LCPUFA. Volatiles started to be formed in hoki liver oil under Condition A from day 5 and from day 15 under Condition B (Figure 4a).

Chemical structures of the measured volatiles (1-penten-3-one, (E)-2-pentenal, 1-penten-3-ol, (E,E)-2,4-heptadienal, and (E,Z)-2,6-nonadienal) are shown in Figure S2A. Higher concentrations of volatiles were measured after 30 days in hoki liver oxidized with Condition B (≈360 mg/kg) compared to Condition A (≈142 mg/kg). The thermal treatment in combination with ambient air had a much stronger influence on volatile levels than sustained exposure to oxygen gas and light, both in hoki liver oil and anchovy oil (Figure 4b). In anchovy oil, volatiles were only formed under Condition B, with the concentration of the five volatiles combined (58.6 mg/kg) being about 1/6th of the concentration measured in hoki liver oil. 1-Penten-3-ol was the most abundant volatile (79.73 ± 1.70 mg/kg) in hoki liver oil at 30 days under Condition A, whereas under Condition B, the dominant volatile was (E,E)-2,4-heptadienal (153.1 ± 1.3 mg/kg) (Table S2). In anchovy oil, the most abundant volatile under Condition B was 1-penten-3-ol (25.7 ± 0.23 mg/kg), whereas under Condition A, only 1-penten-3-one was present, at very low concentrations (0.03 ± 0.00 mg/kg).

### 3.12. Isoprostanoids and Isofuranoids

**Figure 5:**
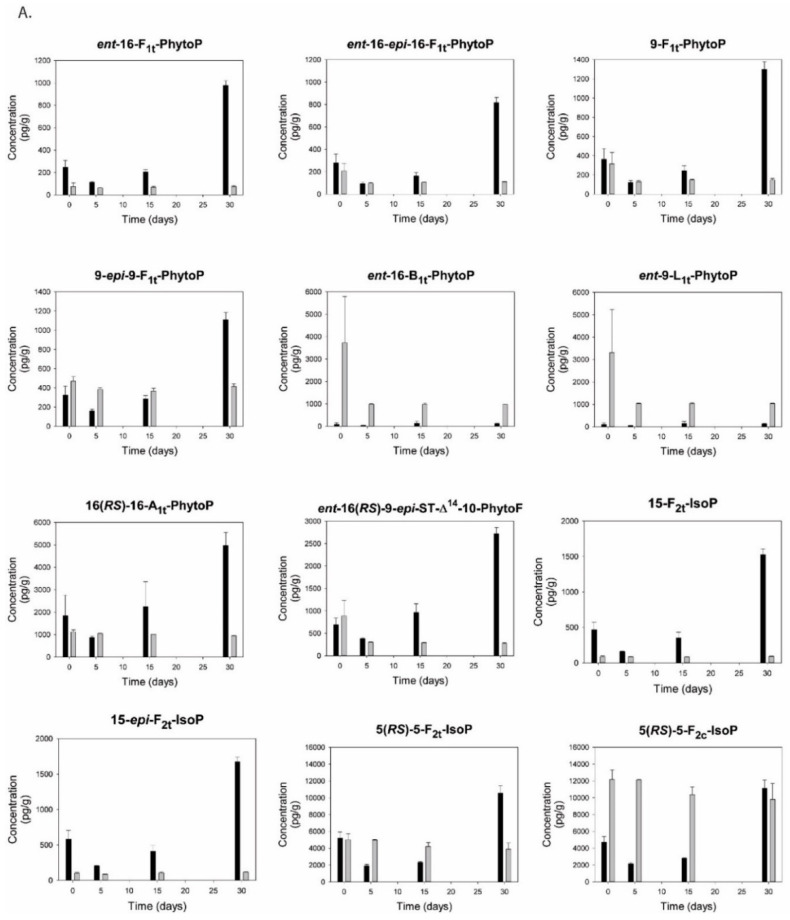

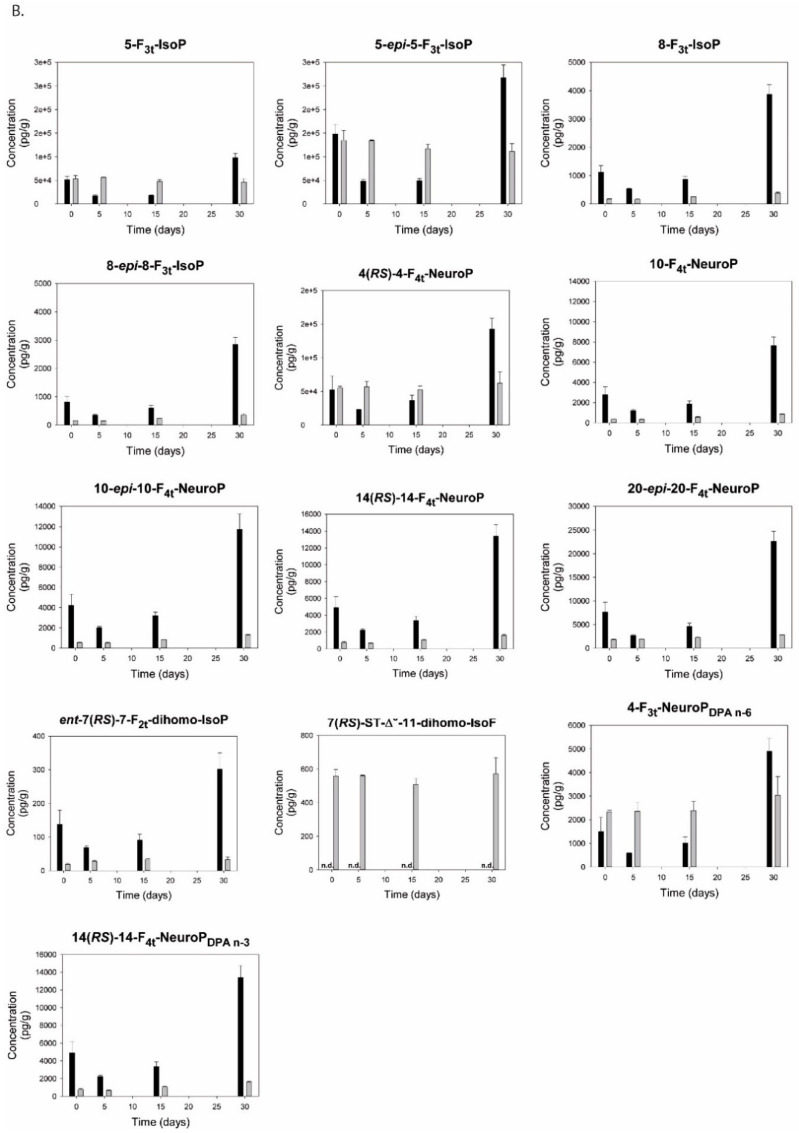
(**A**) Temporal changes of phytoprostanes and arachidonic acid-derived isoprostanes in hoki liver oil (black bars) and anchovy oil (gray bars) during a 30-day incubation under oxidation Condition A. Results are means ± S.D. of three technical replicates for one incubation. (**B**) Temporal changes of EPA-derived isoprostanes, DHA-derived neuroprostanes, adrenic acid (AdA)-derived dihomo-isoprostanes and furans, and DPA n-6- and DPA n-3-derived neuroprostanes in hoki liver oil (black bars) and anchovy oil (gray bars) during a 30-day incubation under oxidation Condition A (oxygen and light exposure at room temperature). Results are means ± S.D. of three technical replicates for one incubation (n.d; not detectable).

In Figure 5A,B, the temporal changes of a range of phytoprostanes, isoprostanes, and neuroprostanes are shown. Chemical structures of the measured isoprostanoids are shown in Figure S2B,C.

In hoki liver oil, the concentrations of several isoprostanoids had markedly increased at 30 days, after remaining relatively stable from baseline to day 15. This was the case for ALA-derived ent-16-F_1t_-PhytoP, ent-16-epi-16-F_1t_-PhytoP, 9-F_1t_-PhytoP, 9-epi-9-F_1t_-PhytoP, 16(RS)-16-A_1t_-PhytoP, as well as for the furanic derivative ent-16(RS)-9-epi-ST-Δ^14^-10-PhytoF (Figure 5A); the arachidonic acid (AA)-derived 15- and 15-epi-isomers of F_2t_-IsoP, the 5(RS)-5-F_2t_-IsoP and the all-cis isomer 5(RS)-5-F_2c_-IsoP (Figure 5A); the EPA-derived 5-F_3t_-IsoP and 5-epi-5-F_3t_-IsoP, the 8-F_3t_-IsoP and 8-epi-F_3t_-IsoP (Figure 5B); the DHA-derived 4-F_3t_-NeuroP, 4(RS)-4-F_4t_-NeuroP, the 10-F_4t_-NeuroP and 10-epi-F_4t_-NeuroP, 14(RS)-14-F_4t_-NeuroP and 20-epi-20-F_4t_-NeuroP (Figure 5B); and the adrenic acid (AdA, C22:4 n-6)-derived ent-7(RS)-7-F_2t_-dihomo-IsoP (Figure 5B), the DPA n-6-derived 4-F_3t_-NeuroP_DPA n-6_, and the DPA n-3-derived 14(RS)-14-F_3t_-NeuroP _DPA n-3_ (Figure 5B). In anchovy oil, the levels of most isoprostanes and neuroprostanes remained stable, while the levels of some of the phytoprostanes decreased within the first few days of the incubation or gradually over the course of the incubation (Figure 5A,B).

5-epi-5-F_3t_-isoprostane (≈180 ng/g oil) and 4(RS)-4-F_4t_-neuroprostane (≈50 ng/g oil) were the most abundant isoprostanoids in both hoki liver oil and in anchovy oil. After 30 days, the levels had increased to ≈320 ng/g and ≈200 ng/g in the hoki liver oil, respectively, while no marked changes had occurred in the anchovy oil. The ALA-derived phytoprostanes ent-9-L_1t_-PhytoP and ent-16-B_1t_-PhytoP were hardly detectable in hoki liver oil but were distinctly present in anchovy oil at baseline (Figure 5A). During the first days of the incubation, the levels of these phytoprostanes decreased ≈75% and thereafter remained at lower levels. The AdA-derived furan 7(RS)-SC-Δ^8^-11-dihomo-IsoF was also unique to anchovy oil, but its level did not change during the incubation (Figure 5B). The ALA-derived phytoprostanes (except 16(RS)-16-A_1t_-PhytoP), AA-derived 15-F_2t_-IsoP, AdA-derived ent-7(RS)-7-F_2t_-dihomo-IsoP and 7(RS)-SC- Δ^8^-11-dihomo-IsoF, and the DPA n-3-derived 14(RS)-14-F_3t_- NeuroP _DPA n-3_ were found in both oil types at relatively low concentrations compared to the products derived from EPA and DHA.

### 3.13. Oxysterols

**Figure 6:**
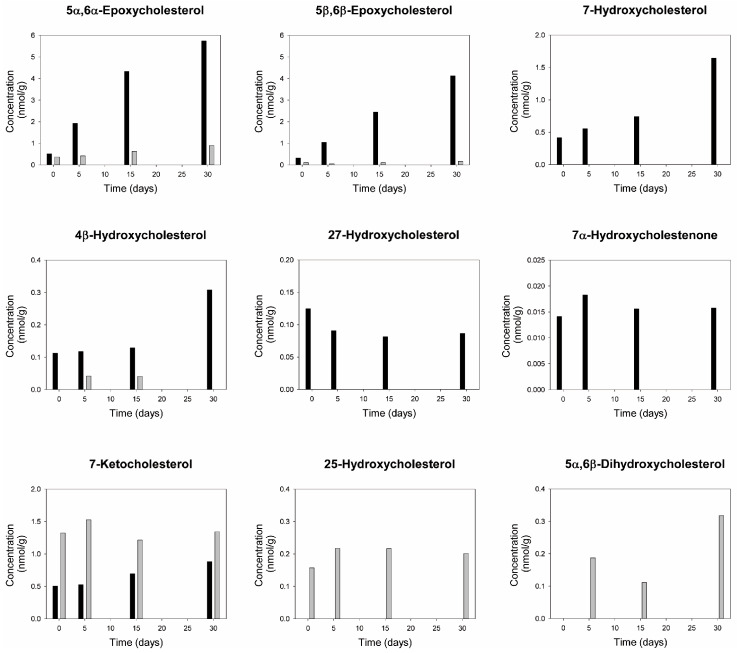
Temporal changes in oxysterols in hoki liver oil (black bars) and anchovy oil (gray bars) during the 30-day incubation under Condition A (exposure to oxygen and light at room temperature). Results are means ± S.D. of three technical replicates for one incubation.

In hoki liver oil, the concentrations of 5α,6α-epoxycholesterol, 5β,6β-epoxycholesterol, and 7-hydroxycholesterol increased continuously throughout the exposure to oxygen and light (Figure 6). Chemical structures of the measured oxysterols are shown in Figure S2D.

The concentrations of 5α,6α-epoxycholesterol and 5β,6β-epoxycholesterol increased approximately 10-fold at day 30 (to ≈5.7 and 4.1 nmol/g oil, respectively) compared to baseline levels. The level of 4β-hydroxycholesterol had increased three-fold at day 30, but its formation was delayed. 7-Ketocholesterol also showed a small but continuous increase during the incubation, although the levels remained below those naturally present in anchovy oil. No remarkable changes in the concentrations of 27-hydroxycholesterol and 7α-hydroxycholestenone were observed. For anchovy oil, no consistent increases in these oxysterols were noted except for 5α,6 α-epoxy-cholesterol, which was formed at low levels in the oil (0.9 nmol/g at day 30). 7-Hydroxycholesterol, 27-hydroxycholesterol, and 7α-hydroxycholestenone were oxysterols only present in hoki liver oil. In contrast, 25-hydroxycholesterol and 5α,6β-dihydroxycholesterol were unique for anchovy oil. 7-Ketocholesterol was present in both oils, and levels only modestly changed during the incubation.

### 3.14. Correlations

In order to determine the strength of the relationship between the various measured chemical parameters and the levels of total tocopherols in the two oils, the correlations between the measured tocopherols and PV, p-AV, induction time, and volatiles for both oxidation conditions were determined by simple linear regression (Table S3). In both hoki liver oil and anchovy oil, PV, p-AV, and volatiles had strong negative correlations (r values ranged from −0.832 to −0.993) with the tocopherol content, while the induction time displayed a strong positive correlation (range 0.774 to 0.997). The correlation between tocopherol content and PV of hoki liver oil under Condition A (oxygen exposure and light at room temperature) was near perfect (r = −0.999). Near perfect correlations were found between tocopherol levels and p-AV (r = −0.999) or volatiles (r = −0.998) under Condition B (elevated temperature without light).

The present study also addressed whether an initial brief (30 s) saturation of the oils with oxygen gas (obtained by a stream of 2.5 L/min for 30 s before placing samples in the oven at 50 °C) would modify the long-term chemical changes (5, 15, and 30 days) under the conditions of Condition B. However, no differences compared to Condition B were seen in PV, p-AV, TOTOX number, induction time, and tocopherol levels (data not shown).

## 4. Discussion

### 4.1. Hoki Liver Oil Over-Oxidation

The present study reproduced the oxidative conditions employed by Albert et al. [28] as well as possible, even though a precise description of the experimental setup, such as the wavelength range and intensity of the illumination used, the rate of oxygen gas delivery, and the geometry and material of the vessel used for the incubation were not reported. By inspecting the temporal profile of PV in the present study, it was nevertheless possible to assess at which degree of oxidation the chemical changes would be approximately similar to those that may have occurred in the original study. Albert et al. reported a PV of 48.8 meq O_2_/kg for the hoki liver oil after one month of accelerated oxidation [28]. The final PV attained in the current study was ≈125 meq O_2_/kg, indicating that the conditions applied in the present study were more intense. In the present study, a similar level of hoki liver oil oxidation was attained at approximately 15 days.

Remarkably, only a relatively small increase in secondary oxidation was found in the study by Albert and colleagues; after 30 days, p-AV had increased from 0.6 to 4.5, which is a value still well within the quality requirements for omega-3 EPA/DHA oils. For comparison, the present study showed a ≈3-fold higher p-AV (13.3) at 15 days and a ≈10-fold higher p-AV (43.8) at 30 days. A range of other indicators, such as oligomers and volatiles, confirmed the marked degree of secondary oxidation that was obtained. One of two plausible reasons may explain the apparently low level of secondary oxidation in Albert et al. [28]. Either fatty acid hydroperoxyl radicals were not effectively further transformed into secondary oxidation products, which is highly improbable, or a substantial portion of the volatile *para*-anisidine-reactive aldehydes formed as secondary oxidation products were stripped out of the incubations more effectively than in the present study. The permanent oxygen gas stream is likely continuously removing a portion of the volatiles formed, suggesting a more intense contact (smaller bubbles) or longer contact time (a more vertically shaped container) of the gas bubbles with the oil in the study by Albert et al.

Both oxidation conditions employed in the present study represent good examples of “open” incubations, in which volatile compounds formed in the oil are easily lost to ambient air as the equilibrium is shifted toward net evaporation [13,14,61]. Since volatile compounds produced during the sustained oxidation incubations can evaporate from the incubations, the pattern of detected volatiles needs to be interpreted as those present in the oils at the moment of sampling. The accumulation of secondary oxidation products with boiling points below ambient temperature (oxidation condition A) or 50 °C (oxidation condition B) will not occur; i.e., they will already have evaporated to a large extent from the oils at the time of sampling. In Condition A, the formation of volatile secondary oxidation products is likely to be underestimated even more, since the continuous oxygen gas stream will strip volatiles out of the incubations faster than the spontaneous evaporation of volatiles from the incubations. This may explain the lower concentrations of volatiles observed in both oil types under Condition A compared to B. This situation does not correspond to the situation of encapsulated or bottled fish oils, which are closed systems without any, or very limited, gas exchange. It is probable though that the extent of secondary oxidation was markedly under-estimated in the study by Albert et al., and that the levels of the secondary oxidation products with low volatility, such as the isoprostanoids and oxysterols, could be present at comparable levels as in the current study at day 15, or between day 15 and 30.

Other small differences in experimental conditions may cause relatively large differences over the long and sustained oxidation period, but their influence is not possible to estimate. Differences in oxygen tension in the oil might also be considered to be a factor, since the rate of lipid peroxidation depends on oxygen tension, as shown for rapeseed oil [62]. Air-saturated water at 20°C contains an oxygen concentration of ≈0.29 mM [63], whereas at full saturation, dissolved oxygen is ≈1.1 mM. The solubility of oxygen in marine triglyceride fish oils is in the range of 1.22–2.44 mmol/kg oil at 20 °C, and it is expected to be higher at 50 °C under Condition B [64]. With continuous oxygen delivery to the oils, oxygen tension is likely maximal, suggesting that a small deviation in the precise rate of continuous delivery of oxygen gas to the oils should not be expected to affect the oxidation rate.

It is clear that a very high level of oxidation was achieved in the hoki liver oil by the photooxidative conditions at high oxygen tension after one month (condition A). The attained TOTOX number of 296 is ≈11 times larger than the maximum limit of 26 set voluntarily by industry and with which many fish oil producers and retailers comply [18]. With the employed antioxidant-free hoki liver oil, this limit was exceeded already within 1 day of incubation. Furthermore, the marked rise in oligomers indicates that the oil was severely damaged, exceeding the pharmacopeial monograph limit of 1.5% that is considered acceptable for refined EPA/DHA omega-3 triglyceride oils [65]. The present study also confirms that massive oxidative damage to an oil, exceeding regulatory quality limits many-fold, is needed to achieve a relatively modest 10% reduction in EPA and DHA content in an unprotected oil, as exemplified by the hoki liver oil in this study. It has previously been shown that the minor oxidative changes that can occur during normal shelf-life conditions of fish oil finished products do not lead to measurable decreases in EPA and DHA content [19]. The content of saturated and monounsaturated fatty acids was not affected, which was expected, since these fatty acids are insensitive, or much less sensitive, to oxidation than PUFA. Other LCPUFA, such as AA, DPA n-3, DPA n-6, and AdA, did not display a decrease in content, but small amounts were nevertheless converted by lipid peroxidation to the corresponding isoprostanoids.

The sustained oxidation of oils and biological membranes rich in PUFA is understood to occur in three stages: initiation, propagation, and termination [13,66]. Initiation involves the formation of fatty acid peroxyl radicals, propagation involves an unimpeded chain reaction of further free radical-mediated lipid peroxidation, and termination forms nonradical products. Once formed, a fatty acid peroxyl radical can abstract a bis-allylic hydrogen atom from another PUFA, thereby forming the corresponding fatty acid hydroperoxide, the major primary oxidation product, and a new PUFA radical. The hydroperoxides can further react with radicals to form alkoxyl radicals or fragment by chain scission, leading to the formation of an array of hydroxylated derivatives, saturated and unsaturated aldehydes, ketones, and short-chain hydrocarbons, which are collectively called secondary oxidation products.

The acid value is sometimes used as an indicator for oxidative quality in fish oils. However, the absence of detectable changes in acid value did not support the hydrolysis of esterified fatty acids to increased free unesterified fatty acids to occur under the experimental conditions. Possibly, the low water content in the oils precluded changes in the acid value. However, hoki liver oil had a three-fold higher baseline acid value (1.49 ± 0.01 mg KOH/g oil) than the anchovy oil (0.49 ± 0.00 mg KOH/g oil), which may be attributable to a higher level of free fatty acids. Carboxylic acids can catalyze the chain scission of fatty acid peroxides, thereby increasing the rate of secondary oxidation of an oil [67]. Although we did not measure the level of free fatty acids directly, it is possible that the more marked development of secondary oxidation in hoki liver oil under thermal oxidation Condition B, compared to the anchovy oil, is promoted by a higher level of free fatty acids.

Another important aspect of reproducing the experimental conditions was that the hoki liver fish oil used in the study by Albert et al. did not contain added antioxidants, whereas the large majority of fish oils, as ingredients and finished products, contain added antioxidants (typically α-tocopherol or mixed tocopherols) to protect the oil from oxidation. This hoki liver oil was minimally refined and still contained some tocopherol (all α-tocopherol, which originates from the liver tissue from which the oil was extracted) but did not contain added antioxidants [68]. The approximately 15-fold lower total tocopherol content than the standard refined and antioxidant-stabilized anchovy oil rendered it much more susceptible to oxidation.

The progress of oxidation in hoki liver oil is well illustrated by the measured changes in induction time, which is a parameter that reflects the remaining resistance of an oil to oxidation. This resistance reflects the level of antioxidant that remains at any time point, which still serves to protect the oil from entering in an uncontrolled radical propagation phase that will consume all available peroxidizable polyunsaturated fat. Tocopherols are chain-breaking antioxidants that donate hydrogen to reduce fatty acid peroxyl radicals and alkoxyl radicals to the corresponding alcohols, lowering the possibility for radical reactions to progress [69,70]. When the induction time had decreased approximately 6-fold, which occurred between day 5 and 15, both primary and secondary oxidation ramped up substantially. Marked increases in PV, p-AV, TOTOX, and oligomers occurred at this time. By day 15, the total tocopherol level was reduced by half and was thereafter completely consumed in the second half of the incubation period. This antioxidant activity was reflected in the near-perfect correlation between induction time and tocopherol content (r = 0.997). In contrast, the formation of total volatiles lagged in terms of the change in induction time compared to the other indicators of secondary oxidation, supporting the idea that a major portion of volatiles is continuously stripped out of the oil by the delivery of oxygen gas to the oil.

### 4.2. Contrasting Distinct Oxidation Conditions

For fish oils and other EPA/DHA omega-3 finished products that consumers purchase, a more realistic way to experience oxidation is exposure to storage conditions that are too hot for the intended formulation but without incident light, since products are usually packaged and bottled in tainted plastic or glass containers that minimize incident light. Exposure to air and light may theoretically occur during fish oil production or encapsulation, but experienced manufacturers generally know how to control this. Nevertheless, it is important to study such different product environments to determine if qualitative and quantitative differences in specific oxidation products may occur. Since the present study looked at two different conditions, it was possible to appreciate that the continuous exposure of hoki liver oil to oxygen gas at room temperature under light conditions led to a much stronger formation of primary oxidation products (fatty acid peroxides) than at elevated temperature (50 °C) in combination with exposure to ambient air in the dark. Between days 2 and 15, secondary oxidation, as measured by p-AV, was stronger under thermal stress in the dark and ambient air exposure, while at day 30, p-AV was similar under both conditions in hoki liver oil. Interestingly, the distribution and absolute levels of specific volatiles was entirely different: at day 15 under Condition A, the volatiles with the highest abundance were 1-penten-3-ol (2.46 ± 0.06 mg/kg) and 1-penten-3-one (1.69 ± 0.02 mg/kg). Under Condition B, the most abundant volatiles were 1-penten-3-ol (124.20 ± 0.90) and (*E*,*E*)-2,4-heptadienal (14.84 ± 0.01 mg/kg). The difference in absolute levels again illustrates the probable accelerated evaporation of volatiles under Condition A, leading to at least a log-order lower level of volatiles under this condition compared to thermal stress in the dark. At day 30, although p-AV was the same under both conditions, the summed level of volatiles that were measured was about 2.5 times higher in Condition B. This indicates that non-volatile aldehydes represented a relatively greater proportion of *para*-anisidine-reactive substances in the hoki liver oil under Condition A than in B. These results show how the pattern of secondary oxidation products is very sensitive to the precise experimental and oxidative conditions.

The finding that a near-perfect negative correlation existed between tocopherol content and PV of hoki liver oil under Condition A (r = −0.993), but less strongly under Condition B, suggests that tocopherols are particularly effective in protecting fish oils from primary oxidation under conditions of photo-oxidation at high oxygen tension. For anchovy oil, this was observed under Condition B for the relationship between tocopherol levels and p-AV (r = −0.999) and volatiles (r = −0.9997), suggesting that tocopherols are excellent protectors of hoki liver oil from secondary oxidation under conditions of elevated temperature in the dark. Thermolysis did not take place under Condition B, since the acid value did not rise.

### 4.3. Comparison of Oxidation in Different Fish Oils

While hoki liver oil is a good source of EPA and DHA, on a global scale, it is used in relatively small volumes compared to other fish oils. Therefore, the susceptibility to oxidation and associated chemical changes of the hoki liver oil were contrasted with the far more commonly used refined anchovy oil. In Condition B, the induction time dropped more gradually over time in hoki liver oil compared to the anchovy oil, which showed drastic decreases in the second half of the incubation. Nevertheless, the induction times attained in the end of the incubation were nearly identical, as were PV, p-AV, and TOTOX, indicating that over the longer period of time, both oils had lost antioxidant protection to the sustained condition of thermal oxidation in the dark (Condition B). The final TOTOX number of 117 meq O_2_/kg (Condition B) is four times larger than the voluntary industry limit of 26, again illustrating the marked rancidity achieved [18]. This result indicates that while at earlier time points the well-stabilized anchovy oil resisted thermal oxidation better than the antioxidant-free hoki liver oil, at longer periods of time, the anchovy oil also quickly lost antioxidant protection.

In contrast, a marked difference in sensitivity to photo-oxidation at high oxygen tension (Condition A) was found between hoki liver and anchovy oil. The anchovy oil proved to be remarkably resistant throughout the 30-day period to this form of oxidative challenge, while the antioxidant-free hoki liver oil became rancid rapidly. While the total polyunsaturated fat content of both oils was comparable, the tocopherol contents were vastly different. The high level of mixed tocopherols present in anchovy oil likely support the delayed occurrence of free radical propagated lipid peroxidation as reflected in reduced primary and ensuing secondary oxidation, as seen in the profile of PV, p-AV, TOTOX, volatiles, and induction time, as compared to hoki liver oil where the antioxidant protection conferred by the naturally present α-tocopherol is considerably lower. The oligomer level in anchovy oil remained below the 1.5% limit set by the European Pharmacopoeia [65], indicating that the oil remained relatively intact even after 30 days. When, after 30 days, the anchovy oil also started to succumb to the strong oxidative environment, tocopherol levels had decreased by only ≈20%. The correct use of mixed tocopherols is an example of how a refined fish oil, which in theory is highly susceptible to oxidation, can be stabilized appreciably through the appropriate use of antioxidants [17]. The level of transition metals was not determined but would provide further information on the sensitivity of the tested oils toward facilitating lipid peroxidation.

### 4.4. Specific Groups of Secondary Oxidation Products and Biological Activity

In the study by Albert et al., about 30% of newborn rat pups, born from dams that received over-oxidized hoki liver oil throughout gestation, had died by day 2 postnatally [28]. Although litter sizes were not affected, no further information on placentation, intra-uterine growth restriction, or other developmental or biochemical alterations during gestation was reported. Pups that did survive had no reported alterations from pups born from dams that had received unoxidized fish oil. Dams that continued to be fed with the rancid hoki liver oil postnatally developed increased insulin resistance at day 22 when offspring was weaned. No further information on dam health during gestation or at parturition was reported. New research is necessary to understand if specific oxidation products formed in oxidized oils that were identified in the present study may be involved in postnatal mortality and other potential perinatal alterations. Specific volatiles, isoprostanoids, and oxysterols have been reported to have potent effects on physiological processes, as discussed next.

#### 4.4.1. Volatiles

Volatiles are frequently measured as an indicator for oxidative quality of marine oils, although no consensus currently exists about which volatiles should be monitored, since the profile can vary depending on oil type and oxidation mechanism. With the exception of 1-penten-3-ol, the volatiles tested in the present study are conjugated aldehydes that will react with the *para*-anisidine reagent and contribute to p-AV value. The formation of unesterified secondary oxidation products originating from esterified LCPUFA, such as various oxygenated *α*,*β*-unsaturated aldehydes, has been shown to occur in thermally treated aerated edible oils [71]. Such conjugated aldehydes are bio-available upon oral intake, and their electrophilicity toward proteins and nucleic acids is linked to cytotoxic and genotoxic activity [72,73]. The reactive aldehydes can react with sulfhydryl groups in proteins, thereby interfering with cellular metabolism and physiological processes. Under Condition A, 1-penten-3-one, (*E*)-2-pentenal, 1-pentene-3-ol, (*E*,*E*)-2,4-heptadienal, and (*E*,*Z*)-2,6-nonadienal were all detectable during the over-oxidation of hoki liver oil at 15 and 30 days. Hence, these volatiles will likely have been present in the oil administered to rats in the study by Albert et al. In a future study, it would therefore be interesting to assess which of these substances, at the concentrations uncovered in this study and administered in correctly allometrically adjusted doses, display perinatal toxicity in pups and dams. To draw meaningful conclusions about the biological risk resulting from exposure to reactive oxidation products, such in vivo studies should also evaluate the dose in relation to endogenous antioxidant defenses and phase 2 metabolism of reactive oxidation products [74,75].

Furthermore, biological activity that is unrelated to electrophilic reactivity should be considered. For example, (*E*)-2-pentenal is a strong irritant of sensory nerves, being a ligand for the TRPA1 channel [76]. Even though these volatiles are highly reactive, they do not show an indiscriminate reactivity, because steric factors are important as well; for example, the *α*,*β*-unsaturated oxo-compounds 1-penten-3-one and (*E*)-2-pentenal inhibit glucose 6-phosphatase, but conjugated dienals do not [77]. Limitations of the present study are recognized, since a wider array of LCPUFA-derived volatiles can be formed in fish oils [78]. The major low molecular weight volatile acrolein, the dialdehyde malonaldehyde, as well as 4-hydroxy-*α*,*β*-unsaturated aldehydes such 4-hydroxy-hexenal, for which cytotoxic effects are documented, were not covered by the present study [78,79]. From the perspective of refined anchovy oil, further research could focus on volatiles formed particularly under conditions of thermal stress in the dark.

#### 4.4.2. Isoprostanoids

The present study reports for the first time that several isoprostanoids in unesterified free form are present in refined fish oils, and that specific isoprostanoids are formed during the development of rancidity. Isoprostanoids, and specifically F_2_-isoprostanes, were originally identified as esterified products within phospholipids in peroxidized biological membranes [80]. Isoprostanoids can also be formed from unesterified omega-3 LCPUFA under peroxidative conditions [81,82]. Given the absence of active deacylating enzymes in refined oils, either the isoprostanoids are released by hydrolysis during oxidation or they are uniquely formed from free fatty acids in the oils (although we do not know what the levels are in this study). It is also possible that isoprostanoids formed during oxidation will be present in esterified form within the triglycerides that make up the bulk of the hoki liver oil and anchovy oil. This will need to be further investigated. Interestingly, all the tested isoprostanoids were also present at baseline, which suggests that these are also formed during the extraction and refining of the oil, and/or they originate from the fish from which the oil was originally extracted. Since isoprostanoids are chemically relatively stable at ambient conditions, it will be interesting to evaluate if specific members could be suitable markers for secondary oxidation in omega-3 EPA/DHA oils, including low-grade oxidation in ingredient oils and finished products. Recently, neuroprostanes derived from DHA and isoprostanes from EPA were shown to be present in two encapsulated marine dietary supplement oils: calanus oil and a fish oil composed of a mixture of sardine, anchovy, mackerel, and herring oil [83].

Several phytoprostanes, isoprostanes, and neuroprostanes identified in the over-oxidized hoki liver oil at day 30 were present at higher levels than before the exposure to oxygen and light or at day 5 or day 15. Several of these substances were also present in considerably higher levels than in anchovy oil. However, strictly taken, at day 15, none of the measured isoprostanoids had increased compared to baseline, which in many cases had initially decreased from baseline to day 5. Between day 5 and day 30, a range of isoprostanoids were formed increasingly, ultimately reaching levels markedly surpassing baseline, typically about 3.5 times. The most abundant isoprostanoids formed from day 5 onwards were 5-*epi*-5-F_3t_-IsoP (0.32 ng/g oil) and 5-F_3t_-IsoP (0.1 ng/g oil), which were both derived from EPA, and the DHA-derived 4(*RS*)-4-F_4t_-NeuroP (0,2 ng/g oil), 20-*epi*-20-F_4t_-NeuroP (0.023 ng/g oil), and 14(*RS*)-14-F_4t_-NeuroP (0.014 ng/g oil). Thus, specific isoprostanoids could be considered of interest to further evaluate to contributing to, or modulating, the postnatal and maternal toxicity as observed by Albert et al.

The abundance of individual PUFA species in each oil was related to the concentrations of isoprostanoids formed. EPA and DHA are clearly a chemical substrate for the formation of specific isoprostanoids, but also other major LCPUFA species present in fish oils, such as AA and ALA, as well as LCPUFA found in lower abundance, such as adrenic acid and DPA n-3, were converted to the corresponding isoprostanes and phytoprostanes (and related furans). In vivo, tissue levels of EPA- and DHA-derived isoprostanes and neuroprostanes increase following dietary supplementation with EPA and DHA [37]. While all the measured isoprostanoids were found in relatively low concentrations, some of these substances have potent biological activity. For example, 15-F_2t_-IsoP has potent vasomotor activity, and it activates platelets [84]. 5-*epi*-5-F_3t_-IsoP regulates neurotransmitter release via activity on the prostanoid EP1 receptor [36]. With regard to the perinatal period, F_2_-isoprostanes are strongly related to the consequences of intra-uterine hypoxia and neonatal morbidity [85]. Elevations in 15-*epi*-15-F_2t_-IsoP and total F_2_-isoprostanes in the cord blood of newborn infants are strongly correlated with the severity of hypoxic–ischemic encephalopathy [86]. At birth, the transition to a higher oxygen tension involves increased oxidative stress, leading to the generation of specific isoprostanoids that are involved in the closure of the ductus arteriosus [87]. The possibility that circulating isoprostanoids derived from a high intake of over-oxidized fish oil may be involved in these processes needs to be investigated.

Although isoprostanoids are useful biomarkers of oxidative stress in organisms, and their formation has been documented in several disorders, their biological activities are varied. The reported activities of EPA-derived isoprostanes and the DHA-derived neuroprostanes are not necessarily negative for health [37]. For example, the 4(*RS*)-4-F_4t_-neuroP and F_3_-IsoPs have anti-arrhythmic and cardioprotective activity [38], and 4(*RS*)-4-F_4t_-NeuroP and 14(*RS*)-14-F_4t_-NeuroP have potent anti-inflammatory activity in macrophages but less than enone A_4_-neuroprostanes [88]. So far, the DHA-derived 4(*RS*)-4-F_4t_-NeuroP is the most potent LCPUFA-derived isoprostanoid identified and possesses antiarrhythmic activities both in vivo and in vitro via the protection of the ryanodine receptor [89] and similarly protects against ventilator-induced diaphragmatic dysfunction in vivo [90]. Recently, Lee et al. reported that 4(*RS*)-4-F_4t_-NeuroP upregulates the transcriptional level of the antioxidant enzyme heme oxygenase-1 in SH-SY5Y cells and primary neuronal culture, confirming the bioactivity of this neuroprostane [91].

#### 4.4.3. Oxysterols

We also addressed if oxysterols might be formed in hoki liver oil during the prolonged over-oxidation induced by exposure to oxygen and light. Three oxysterols, 5α,6α-epoxycholesterol, 5β,6β-epoxycholesterol, and 7-hydroxycholesterol, proved to be very sensitive markers of the oxidation process, showing increases in level already at day 5. That these correspond to two epoxides and a primary alcohol suggests that these oxysterols reflect primary oxidation, which is analogous to the formation of fatty acid hydroperoxides. Interestingly, the two 5,6-epoxy derivatives, which are highly sensitive to oxidative conditions, were also the most abundant oxysterols detected in the oil after the oxidation process.

We did not determine the content of cholesterol of the oils used in the present study. However, the level of cholesterol in refined hoki liver oil has been reported to be 1.41 g/kg [92]. Refined anchovy oil that has been refined, bleached, deodorized, and further processed by molecular distillation contains cholesterol in the range of 3–6 g/kg [93]. Thus, the levels of cholesterol in these two refined oils are likely to be present in a similar concentration range, and our initial hypothesis that cholesterol level would be substantially higher in hoki liver oil than in anchovy oil does not hold. Thus, the availability of cholesterol as an oxidizable substrate is not the explanation for the higher formation of oxysterols in hoki liver oil than in the anchovy oil, although understanding the relative contributions of esterified and free cholesterol to oxysterol formation merits further research. The main difference remains the large difference in susceptibility to oxidation, which is mainly defined by the difference in antioxidant protection. Antioxidants such as tocopherols are able to reduce the levels of oxysterols in a variety of foods [94]. The finding that specific oxysterols can be formed during the over-oxidation of fish oil has not been reported previously. Nevertheless, the levels are low (nmol per kg oil range). Interestingly, both unoxidized hoki liver oil and anchovy oil already contained low levels of various oxysterols, some of which are unique to each oil type.

The formation of oxysterols has been associated with the onset and progression of a range of disorders [95,96]. The pathophysiological activities of oxysterols are inter-related with the multiple biological functions of cholesterol, including being a precursor molecule for the synthesis of steroid hormones, neuroactive steroids, and bile acids. Many oxysterols are cytotoxic toward endothelial and smooth muscle cells at low micromolar concentrations, including 5,6-epoxycholesterol and 7-hydroxycholesterol, which increased markedly in the over-oxidized hoki liver oil [97,98,99]. The addition of oxidized cholesterol to salmon oil has been shown to place a marked stress on antioxidant defenses in rat liver [100]. Oxysterols may also mediate compromised insulin sensitivity, resulting from the consumption of cholesterol-rich diets, as well as toxic effects on the central nervous system [101,102]. Several oxysterols, such as 25-hydroxycholesterol, 7-ketocholesterol, and 22-hydroxycholesterol, can impair the differentiation and fusion of term trophoblast cells and alter their secretion of chorionic gonadotrophin, via activating nuclear liver X receptors (LXR) and inhibition of Sterol Response Element Binding Protein-2 (SREBP-2), which may have important implications for placental development and function [103,104]. Although specific research related to perinatal morbidity has not been carried out, there are multiple possibilities regarding how specific oxysterols might compromise the health of rat pups and dams following the exposure to excessive amounts of dietary oxysterols.

## 5. Conclusions

The temporal analysis of chemical compositional changes of minimally refined antioxidant-free hoki liver oil under harsh oxidative conditions has confirmed the marked over-oxidation of the oil. Extension of the study to a different type of oxidation condition, as well as including a more commonly used and antioxidant-stabilized refined anchovy oil, demonstrated that different oils contain and develop distinct patterns of oxidation products, and that the presence of added antioxidants markedly delays oxidation. This study shows that besides fatty acid peroxides, other oxidation products need to be considered to understand the biological activities of any oxidized fish oil or EPA/DHA-containing product. Furthermore, oxidation products that originate from oil components other than EPA and DHA should also be taken into account, as each specific oil type also contain other fatty acids and lipids that are susceptible to oxidation and that could potentially contribute to the observed biological outcome. It is not appropriate to draw conclusions for all fish oil ingredients and products based on the results obtained with one specific fish oil ingredient or product.

It is important to emphasize that under production and retail conditions, fish oils and other EPA/DHA omega-3 products would never be exposed to the harshness of the employed experimental oxidation conditions in this study. Furthermore, finished products are typically stabilized with added antioxidants and encapsulated or bottled and frequently packaged in a carton box, which provides resistance to oxidation. The relevance of the present study’s findings is only understood in the context of repeating the study by Albert et al. as closely as possible [28]. In addition to the imposition of an unrealistically severe oxidation condition, a further concern is that the 1 mL daily dose of over-oxidized hoki liver oil that was administered to the pregnant rats is too large. Allometric scaling based on body surface area indicates that 1 mL oil (estimated density 0.95 g/mL) given to a rat of 113 days (≈300 g) would be ≈46 mL oil to an adult (60 kg) person, which is more than one log-order of magnitude larger than a typical daily supplement dose [105]. A lower gavage volume is certainly possible, and a 50-fold dilution of the oxidized oil, for example with an unoxidized oil, would have been appropriate. While the authors did state that the results obtained in rats cannot be extrapolated to humans, it was implied that oxidized fish oils would affect newborns and the mother. It was previously pointed out that an improved toxicological assessment should be carried out to determine the effect of over-oxidized hoki liver oil on perinatal health [106].

This does not mean that the results with over-oxidized oils are irrelevant. First of all, we uncovered that both unoxidized fish oils already contained a background level of a particular set of isoprostanoids and oxysterols, which may be naturally present in fish and/or are formed during processing and/or storage of the oils. The baseline levels of these oxysterols are low, suggesting that these do not have adverse effects even at the high dosing used in the study by Albert et al. Several volatiles, isoprostanoids, and oxysterols are formed specifically in the over-oxidized hoki liver oil, which constitute interesting candidates to further assess for their toxicological effects. It is of interest to determine which of these oxidation products have the highest biological activities and are least effectively metabolized and cleared by endogenous detoxification systems in the body. Once these products are identified, a more serious risk assessment can be carried out. Given that the larger fraction of volatiles was likely removed by the oxygen gas delivery, this study indicates that a stronger focus should be placed on understanding the products with low volatility, such as some of the isoprostanoids and oxysterols that are formed during oxidation, to understand the perinatal effects of over-oxidized hoki liver oil observed in the study by Albert et al. Further exploration of specific isoprostanes, neuroprostanes or oxysterols identified in this study may also be of interest in identifying suitable chemically stable markers of primary and secondary oxidation in marine oils, EPA/DHA concentrates, and other omega-3 LCPUFA-rich oils such as krill and algae oils.

This study shows that understanding of the chemical composition of fish oils during rancidity development can improve the interpretation of in vivo studies carried out with oxidized oils. While this study covered a wide range of aspects of oil oxidation, the analysis of chemical changes was certainly not exhaustive. Limitations in the experimental oxidation conditions preclude making general statements on the oxidation pathways that will occur in different oil types and formulations produced and stored under different environments. The results of oxidized fish oil affecting biology after their administration, as well as the detailed oil composition in relation to the conditions under which the oil deteriorated, should be communicated with as much precision as possible. Future studies may reveal if similar or additional bioactive oxidation products, such as isoprostanoids and oxysterols, are also present in fish oils of good quality, as well as those close to and just above current regulatory and voluntary industry quality limits. Then, such information can be complemented with in vivo studies that are informed about the precise molecular compositions of the oils under realistic conditions of consumption.

## Supplementary Materials

The following are available online at https://www.mdpi.com/2304-8158/9/10/1501/s1, Figure S1: Acid value in hoki liver oil (panel A) and anchovy oil (panel B) during a 30-day incubation under oxidation Condition A (black bars) and B (gray bars), Figure S2A: Chemical structures of volatiles, Figure S2B: Chemical structures of ALA-derived phytofurans and AA-derived isoprostanes. The fatty acid from which the isoprostanoid is derived is given below the substance name, Figure S2C: Chemical structures of EPA-derived isoprostanes, DHA-derived neuroprostanes, adrenic acid-derived and DPA n-6- and DPA n-3-derived isoprostanoids. The fatty acid from which the isoprostanoid is derived is given below the substance name, Figure S2D: Chemical structures of oxysterols, Table S1: Fatty acid composition (in area %) of hoki liver oil and anchovy oil during the 30-day incubation under oxidation conditions A and B, Table S2: Concentrations of volatiles (mg/kg) in hoki liver oil and anchovy oil during the 30-day incubation under oxidation Conditions A and B, Table S3: Correlation analysis between tocopherol concentrations and oxidation parameters.
