# Supplementary material for: Chemical Compositional Changes in Over-Oxidized Fish Oils
Source: Foods. 2020 Oct 20;9(10):1501. doi: 10.3390/foods9101501 (PMC7590219; doi:10.3390/foods9101501)
Supplement: Supplementary file 1 [file foods-09-01501-s001.zip › untitled folder/Fig S2D.docx]

**Fig. S2D**
