# Supplementary material for: Chemical Compositional Changes in Over-Oxidized Fish Oils
Source: Foods. 2020 Oct 20;9(10):1501. doi: 10.3390/foods9101501 (PMC7590219; doi:10.3390/foods9101501)
Supplement: Supplementary file 1 [file foods-09-01501-s001.zip › untitled folder/Table S1.docx]

**Table S1.**

|  |  | Hoki liver oil | | Anchovy oil | |
| --- | --- | --- | --- | --- | --- |
| Fatty acid | **Time point**  **(days)** | **Condition A** | **Condition B** | **Condition A** | **Condition B** |
| C12:0 | 0 | n.d. | n.d. | 0.1 ± 0.0 | 0.1 ± 0.0 |
|  | 5 | n.d. | n.d. | 0.1 ± 0.0 | 0.1 ± 0.0 |
|  | 15 | n.d. | n.d. | 0.1 ± 0.0 | 0.1 ± 0.0 |
|  | 30 | n.d. | n.d. | 0.1 ± 0.0 | 0.1 ± 0.0 |
| C14:0 | 0 | 3.9 ± 0.0 | 3.8 ± 0.0 | 7.1 ± 0.0 | 7.1 ± 0.0 |
|  | 5 | 3.9 ± 0.0 | 3.8 ± 0.0 | 7.1 ± 0.0 | 7.1 ± 0.0 |
|  | 15 | 3.9 ± 0.0 | 3.9 ± 0.0 | 7.1 ± 0.0 | 7.1 ± 0.0 |
|  | 30 | 3.9 ± 0.0 | 3.9 ± 0.0 | 7.1 ± 0.0 | 7.2 ± 0.0 |
| C14:1 n-5 | 0 | 0.2 ± 0.0 | 0.2 ± 0.0 | 0.3 ± 0.0 | 0.3 ± 0.0 |
|  | 5 | 0.2 ± 0.0 | 0.2 ± 0.0 | 0.3 ± 0.0 | 0.3 ± 0.0 |
|  | 15 | 0.2 ± 0.0 | 0.2 ± 0.0 | 0.3 ± 0.0 | 0.3 ± 0.0 |
|  | 30 | 0.2 ± 0.0 | 0.2 ± 0.0 | 0.3 ± 0.0 | 0.3 ± 0.0 |
| C15:0 | 0 | 0.5 ± 0.0 | 0.5 ± 0.0 | 0.8 ± 0.0 | 0.8 ± 0.0 |
|  | 5 | 0.5 ± 0.0 | 0.5 ± 0.0 | 0.8 ± 0.0 | 0.8 ± 0.0 |
|  | 15 | 0.5 ± 0.0 | 0.5 ± 0.0 | 0.8 ± 0.0 | 0.8 ± 0.0 |
|  | 30 | 0.5 ± 0.0 | 0.5 ± 0.0 | 0.8 ± 0.0 | 0.8 ± 0.0 |
| C15:1 n-5 | 0 | 0.2 ± 0.0 | 0.2 ± 0.0 | 0.2 ± 0.0 | 0.2 ± 0.0 |
|  | 5 | 0.2 ± 0.0 | 02 ± 0.0 | 0.2 ± 0.0 | 0.2 ± 0.0 |
|  | 15 | 0.2 ± 0.0 | 0.2 ± 0.0 | 0.2 ± 0.0 | 0.2 ± 0.0 |
|  | 30 | 0.2 ± 0.0 | 0.2 ± 0.0 | 0.2 ± 0.0 | 0.2 ± 0.0 |
| C16:0 | 0 | 15.5 ± 0.1 | 15.4 ± 0.0 | 18.5 ± 0.0 | 18.5 ± 0.1 |
|  | 5 | 15.6 ± 0.1 | 15.4 ± 0.0 | 18.5 ± 0.1 | 18.5 ± 0.0 |
|  | 15 | 15.6 ± 0.0 | 15.5 ± 0.0 | 18.4 ± 0.0 | 18.6 ± 0.1 |
|  | 30 | 15.8 ± 0.0 | 15.6 ± 0.0 | 18.3 ± 0.1 | 18.8 ± 0.0 |
| C16:1 n-7 | 0 | 5.9 ± 0.0 | 5.9 ± 0.0 | 6.6 ± 0.0 | 7.0 ± 0.0 |
|  | 5 | 5.9 ± 0.1 | 5.8 ± 0.0 | 6.6 ± 0.0 | 6.9 ± 0.0 |
|  | 15 | 5.6 ± 0.0 | 5.9 ± 0.0 | 6.6 ± 0.0 | 7.0 ± 0.0 |
|  | 30 | 5.9 ± 0.0 | 5.9 ± 0.0 | 6.7 ± 0.0 | 7.1 ± 0.0 |
| C17:0 | 0 | 0.2 ± 0.0 | 0.2 ± 0.0 | 0.7 ± 0.0 | 0.7 ± 0.0 |
|  | 5 | 0.2 ± 0.0 | 0.2 ± 0.0 | 0.7 ± 0.0 | 0.7 ± 0.0 |
|  | 15 | 0.2 ± 0.0 | 0.2 ± 0.0 | 0.7 ± 0.0 | 0.7 ± 0.0 |
|  | 30 | 0.2 ± 0.0 | 0.2 ± 0.0 | 0.7 ± 0.0 | 0.7 ± 0.0 |
| C17:1 n-7 | 0 | n.d. | n.d. | 0.1 ± 0.0 | 0.1 ± 0.0 |
|  | 5 | n.d. | n.d. | 0.1 ± 0.0 | 0.1 ± 0.0 |
|  | 15 | n.d. | n.d. | 0.1 ± 0.0 | 0.1 ± 0.0 |
|  | 30 | n.d. | n.d. | 0.1 ± 0.0 | n.d. |
| C18:0 | 0 | 2.2 ± 0.0 | 2.2 ± 0.0 | 4.1 ± 0.0 | 4.1 ± 0.0 |
|  | 5 | 2.2 ± 0.0 | 2.2 ± 0.0 | 4.1 ± 0.0 | 4.1 ± 0.0 |
|  | 15 | 2.2 ± 0.0 | 2.2 ± 0.0 | 4.1 ± 0.0 | 4.2 ± 0.0 |
|  | 30 | 2.2 ± 0.0 | 2.2 ± 0.0 | 4.1 ± 0.0 | 4.2 ± 0.0 |
| C18:1 n-9 | 0 | 25.5 ± 0.2 | 23.2 ± 0.0 | 11.4 ± 0.0 | 11.6 ± 0.1 |
|  | 5 | 25.5 ± 0.1 | 23.2 ± 0.0 | 11.4 ± 0.0 | 11.7 ± 0.0 |
|  | 15 | 23.1 ± 0.0 | 23.3 ± 0.1 | 11.4 ± 0.0 | 11.8 ± 0.1 |
|  | 30 | 23.6 ± 0.1 | 23.5 ± 0.2 | 11.5 ± 0.0 | 11.9 ± 0.0 |
| C18:2 n-6 | 0 | 1.0 ± 0.0 | 1.0 ± 0.0 | 1.7 ± 0.0 | 1.7 ± 0.0 |
|  | 5 | 1.0 ± 0.0 | 1.0 ± 0.0 | 1.7 ± 0.0 | 1.7 ± 0.0 |
|  | 15 | 1.0 ± 0.0 | 1.0 ± 0.0 | 1.7 ± 0.0 | 1.7 ± 0.0 |
|  | 30 | 1.0 ± 0.0 | 1.0 ± 0.0 | 1.7 ± 0.0 | 1.7 ± 0.0 |
| C18:3 n-3 | 0 | 0.1 ± 0.0 | 0.1 ± 0.0 | 0.4 ± 0.0 | 0.4 ± 0.0 |
|  | 5 | 0.1 ± 0.0 | 0.1 ± 0.0 | 0.4 ± 0.0 | 0.4 ± 0.0 |
|  | 15 | 0.1 ± 0.0 | 0.1 ± 0.0 | 0.4 ± 0.0 | 0.4 ± 0.0 |
|  | 30 | 0.1 ± 0.0 | 0.1 ± 0.0 | 0.4 ± 0.0 | 0.4 ± 0.0 |
| C18:3 n-6 | 0 | 0.4 ± 0.0 | 0.4 ± 0.0 | 1.1 ± 0.0 | 1.0 ± 0.0 |
|  | 5 | 0.4 ± 0.0 | 0.4 ± 0.0 | 1.0 ± 0.0 | 1.0 ± 0.0 |
|  | 15 | 0.4 ± 0.0 | 0.4 ± 0.0 | 1.0 ± 0.0 | 1.0 ± 0.0 |
|  | 30 | 0.4 ± 0.0 | 0.4 ± 0.0 | 1.0 ± 0.0 | 1.0 ± 0.0 |
| C20:0 | 0 | 0.1 ± 0.0 | 0.1 ± 0.0 | 0.7 ± 0.0 | 0.7 ± 0.0 |
|  | 5 | 0.1 ± 0.0 | 0.1 ± 0.0 | 0.6 ± 0.0 | 0.7 ± 0.0 |
|  | 15 | 0.1 ± 0.0 | 0.1 ± 0.0 | 0.7 ± 0.0 | 0.7 ± 0.0 |
|  | 30 | 0.1 ± 0.0 | 0.1 ± 0.0 | 0.6 ± 0.0 | 0.7 ± 0.0 |
| C20:1 n-9 | 0 | 9.9 ± 0.6 | 10.0 ± 0.0 | 0.2 ± 0.0 | 0.2 ± 0.0 |
|  | 5 | 9.9 ± 0.6 | 0.4 ± 0.0 | 0.2 ± 0.0 | 0.2 ± 0.0 |
|  | 15 | 10.3 ± 0.0 | 0.4 ± 0.0 | 0.2 ± 0.0 | 0.2 ± 0.0 |
|  | 30 | 10.2 ± 0.0 | 10.1 ± 0.0 | 0.2 ± 0.0 | 0.2 ± 0.0 |
| C20:2 n-6 | 0 | 0.3 ± 0.0 | 0.3 ± 0.0 | 0.2 ± 0.0 | 0.2 ± 0.0 |
|  | 5 | 0.3 ± 0.0 | 0.3 ± 0.0 | 0.2 ± 0.0 | 0.2 ± 0.0 |
|  | 15 | 0.3 ± 0.0 | 0.3 ± 0.0 | 0.2 ± 0.0 | 0.2 ± 0.0 |
|  | 30 | 0.3 ± 0.0 | 0.3 ± 0.0 | 0.2 ± 0.0 | 0.2 ± 0.0 |
| C20:3 n-3 | 0 | 0.2 ± 0.0 | 0.2 ± 0.0 | 0.3 ± 0.0 | 0.3 ± 0.0 |
|  | 5 | 0.2 ± 0.0 | 0.2 ± 0.0 | 0.2 ± 0.0 | 0.3 ± 0.0 |
|  | 15 | 0.2 ± 0.0 | 0.2 ± 0.0 | 0.3 ± 0.0 | 0.3 ± 0.0 |
|  | 30 | 0.2 ± 0.0 | 0.2 ± 0.0 | 0.3 ± 0.0 | 0.3 ± 0.0 |
| C20:3 n-6 | 0 | 0.2 ± 0.0 | 0.2 ± 0.0 | 0.1 ± 0.0 | 0.1 ± 0.0 |
|  | 5 | 0.2 ± 0.0 | 0.2 ± 0.0 | 0.1 ± 0.0 | 0.1 ± 0.0 |
|  | 15 | 0.2 ± 0.0 | 0.2 ± 0.0 | 0.1 ± 0.0 | 0.1 ± 0.0 |
|  | 30 | 0.2 ± 0.0 | 0.2 ± 0.0 | 0.1 ± 0.0 | 0.1 ± 0.0 |
| C22:0 | 0 | 4.7 ± 0.0 | 6.1 ± 0.0 | 12.6 ± 0.0 | 12.7 ± 0.0 |
|  | 5 | 4.7 ± 0.0 | 6.0 ± 0.0 | 12.6 ± 0.0 | 12.6 ± 0.0 |
|  | 15 | 4.0 ± 0.0 | 6.0 ± 0.0 | 12.6 ± 0.1 | 12.4 ± 0.1 |
|  | 30 | 4.1 ± 0.0 | 5.9 ± 0.0 | 12.7 ± 0.0 | 12.4 ± 0.0 |
| C22:1 n-9 | 0 | 0.1 ± 0.0 | 0.1 ± 0.0 | 0.2 ± 0.0 | 0.2 ± 0.0 |
|  | 5 | 0.1 ± 0.0 | 0.1 ± 0.0 | 0.2 ± 0.0 | 0.2 ± 0.1 |
|  | 15 | 0.1 ± 0.0 | 0.1 ± 0.0 | 0.2 ± 0.0 | 0.2 ± 0.0 |
|  | 30 | 0.1 ± 0.0 | 0.1 ± 0.0 | 0.1 ± 0.0 | 0.2 ± 0.0 |
| C22:6 n-3 | 0 | 12.4 ± 0.1 | 12.3 ± 0.0 | 15.1 ± 0.0 | 15.2 ± 0.0 |
|  | 5 | 12.3 ± 0.1 | 12.2 ± 0.1 | 14.9 ± 0.1 | 15.3 ± 0.0 |
|  | 15 | 12.5 ± 0.0 | 12.1 ± 0.0 | 15.1 ± 0.1 | 15.0 ± 0.0 |
|  | 30 | 11.5 ± 0.0 | 11.9 ± 0.0 | 15.2 ± 0.0 | 14.9 ± 0.0 |
