# Supplementary material for: Chemical Compositional Changes in Over-Oxidized Fish Oils
Source: Foods. 2020 Oct 20;9(10):1501. doi: 10.3390/foods9101501 (PMC7590219; doi:10.3390/foods9101501)
Supplement: Supplementary file 1 [file foods-09-01501-s001.zip › untitled folder/Table S2.docx]

**Table S2.**

| **Hoki Liver Oil** | | | | **Anchovy Oil** | | |
| --- | --- | --- | --- | --- | --- | --- |
| Volatile | Time point (days) | Condition A | Condition B | Condition A | Condition B |  |
| 1-penten-3-one | 0 | 0.34 ± 0.01 | 0.38 ± 0.02 | n.d. | n.d. |  |
|  | 5 | 0.42 ± 0.02 | 2.26 ± 0.25 | 0.01 ± 0.00 | 0.08 ± 0.00 |  |
|  | 15 | 1.69 ± 0.02 | 11.23 ± 0.74 | 0.02 ± 0.00 | 1.06 ± 0.11 |  |
|  | 30 | 35.29 ± 5.50 | 60.84 ± 0.51 | 0.03 ± 0.00 | 13.06 ± 0.75 |  |
| (*E*)-2-pentenal | 0 | 0.07 ± 0.00 | 0.08 ± 0.00 | n.d. | n.d. |  |
|  | 5 | 0.04 ± 0.00 | 0.80 ± 0.05 | n.d. | n.d. |  |
|  | 15 | 0.12 ± 0.02 | 5.30 ± 0.36 | n.d. | 0.12 ± 0.00 |  |
|  | 30 | 6.75 ± 0.12 | 27.41 ± 0.16 | n.d. | 4.19 ± 0.17 |  |
| 1-penten-3-ol | 0 | 0.81 ± 0.05 | 0.67 ± 0.03 | n.d. | n.d. |  |
|  | 5 | 0.83 ± 0.04 | 6.14 ± 0.38 | n.d. | 0.05 ± 0.00 |  |
|  | 15 | 2.46 ± 0.06 | 26.52 ± 0.64 | n.d. | 3.36 ± 0.28 |  |
|  | 30 | 79.73 ± 1.70 | 124.20 ± 0.90 | n.d. | 25.70 ± 0.23 |  |
| (*E*,*E*)-2,4-heptadienal | 0 | 0.14 ± 0.02 | 0.21 ± 0.01 | n.d. | n.d. |  |
|  | 5 | 0.15 ± 0.01 | 1.51 ± 0.07 | n.d. | 0.01 ± 0.00 |  |
|  | 15 | 0.76 ± 0.01 | 14.84 ± 0.01 | n.d. | 0.32 ± 0.01 |  |
|  | 30 | 19.38 ± 1.48 | 153.11 ± 1.29 | n.d. | 15.51 ± 0.48 |  |
| (*E*,*Z*)-2,6-nonadienal | 0 | 0.01 ± 0.00 | 0.01 ± 0.00 | n.d. | n.d. |  |
|  | 5 | 0.01 ± 0.00 | 0.02 ± 0.00 | n.d. | n.d. |  |
|  | 15 | 0.04 ± 0.00 | 0.10 ± 0.01 | n.d. | 0.01 ± 0.00 |  |
|  | 30 | 0.55 ± 0.12 | 0.86 ± 0.02 | n.d. | 0.17 ± 0.00 |  |
| n.d.: not detected | | | | | | |
