# Supplementary material for: Chemical Compositional Changes in Over-Oxidized Fish Oils
Source: Foods. 2020 Oct 20;9(10):1501. doi: 10.3390/foods9101501 (PMC7590219; doi:10.3390/foods9101501)
Supplement: Supplementary file 1 [file foods-09-01501-s001.zip › untitled folder/Table S3.docx]

**Table S3.**

|  |  | Hoki liver oil | Anchovy oil |
| --- | --- | --- | --- |
| Oxidation Condition | Chemical  Parameter | Linear correlation w/  tocopherol content (r) | Linear correlation w/  Tocopherol content (r) |
| A | PV | -0.993 | -0.874 |
|  | p-AV | -0.963 | -0.940 |
|  | Induction Time | 0.763 | 0.882 |
|  | Volatiles | -0.895 | -0.832 |
|  |  |  |  |
| B | PV | -0.943 | -0.890 |
|  | p-AV | -0.797 | -0.999 |
|  | Induction Time | 0.997 | 0.827 |
|  | Volatiles | -0.612 | -0.998 |
