# Supplementary figures and images for: Chemical Compositional Changes in Over-Oxidized Fish Oils
Source: Foods. 2020 Oct 20;9(10):1501. doi: 10.3390/foods9101501 (PMC7590219; doi:10.3390/foods9101501)

**Fig. S2B**

**
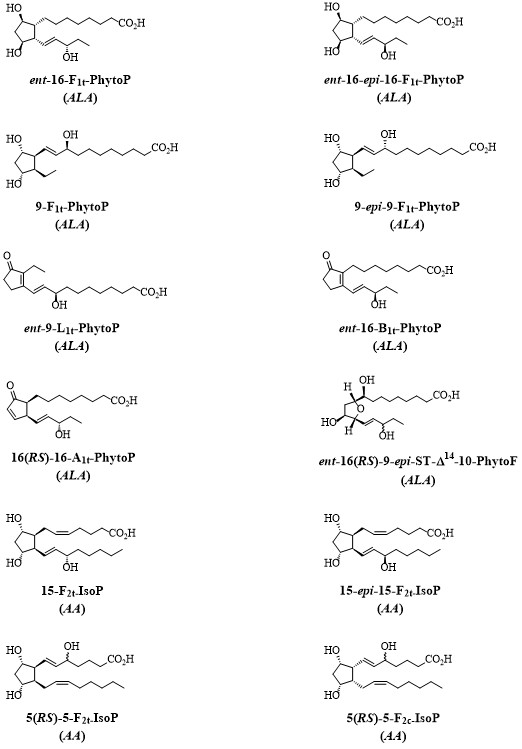
**

Supplement: Supplementary file 1 [file foods-09-01501-s001.zip › untitled folder/Fig S2B.docx]

**Fig. S2C**

**
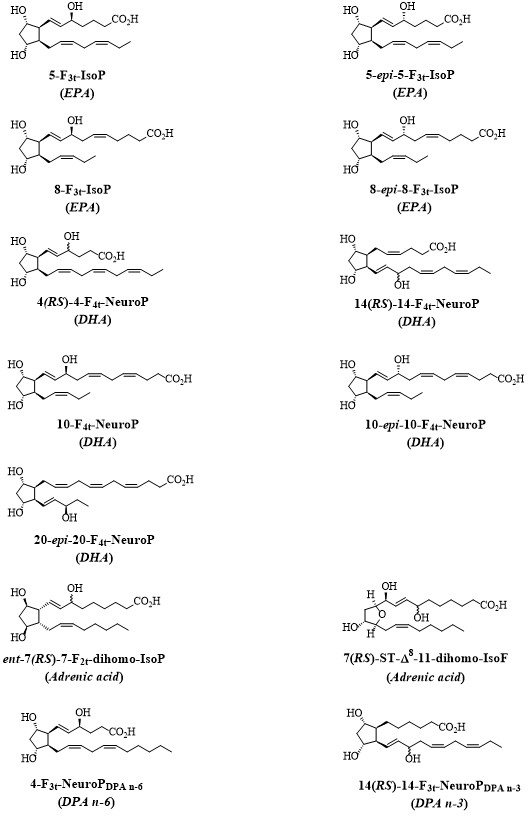
**

Supplement: Supplementary file 1 [file foods-09-01501-s001.zip › untitled folder/Fig S2C.docx]
